# hMRI – A toolbox for quantitative MRI in neuroscience and clinical research

**DOI:** 10.1016/j.neuroimage.2019.01.029

**Published:** 2019-07-01

**Authors:** Karsten Tabelow, Evelyne Balteau, John Ashburner, Martina F. Callaghan, Bogdan Draganski, Gunther Helms, Ferath Kherif, Tobias Leutritz, Antoine Lutti, Christophe Phillips, Enrico Reimer, Lars Ruthotto, Maryam Seif, Nikolaus Weiskopf, Gabriel Ziegler, Siawoosh Mohammadi

**Affiliations:** a WIAS Berlin, Germany; b GIGA Institute, University of Liège, Liège, Belgium; c Wellcome Centre for Human Neuroimaging, London, UK; d Laboratory for Research in Neuroimaging, Department of Clinical Neuroscience, Lausanne University Hospital and University of Lausanne, Switzerland; e Department of Neurology, Max Planck Institute for Human Cognitive and Brain Sciences, Leipzig, Germany; f Medical Radiation Physics, Department of Clinical Sciences Lund, Lund University, Lund, Sweden; g Department of Neurophysics, Max Planck Institute for Human Cognitive and Brain Sciences, Leipzig, Germany; h Emory University, Atlanta, USA; i University of Zurich, Switzerland; j Institute for Cognitive Neurology and Dementia Research, University of Magdeburg, Germany; k Medical Center Hamburg-Eppendorf, Hamburg, Germany

**Keywords:** Quantitative MRI, In vivo histology, Microstructure, Multi-parameter mapping, Relaxometry, SPM toolbox

## Abstract

Neuroscience and clinical researchers are increasingly interested in quantitative magnetic resonance imaging (qMRI) due to its sensitivity to micro-structural properties of brain tissue such as axon, myelin, iron and water concentration. We introduce the hMRI-toolbox, an open-source, easy-to-use tool available on GitHub, for qMRI data handling and processing, presented together with a tutorial and example dataset. This toolbox allows the estimation of high-quality multi-parameter qMRI maps (longitudinal and effective transverse relaxation rates $R_{1}$ and $R_{2}^{⋆}$, proton density PD and magnetisation transfer MT saturation) that can be used for quantitative parameter analysis and accurate delineation of subcortical brain structures. The qMRI maps generated by the toolbox are key input parameters for biophysical models designed to estimate tissue microstructure properties such as the MR g-ratio and to derive standard and novel MRI biomarkers. Thus, the current version of the toolbox is a first step towards *in vivo* histology using MRI (hMRI) and is being extended further in this direction. Embedded in the Statistical Parametric Mapping (SPM) framework, it benefits from the extensive range of established SPM tools for high-accuracy spatial registration and statistical inferences and can be readily combined with existing SPM toolboxes for estimating diffusion MRI parameter maps. From a user's perspective, the hMRI-toolbox is an efficient, robust and simple framework for investigating qMRI data in neuroscience and clinical research.

## Graphical abstract

**Figure:**
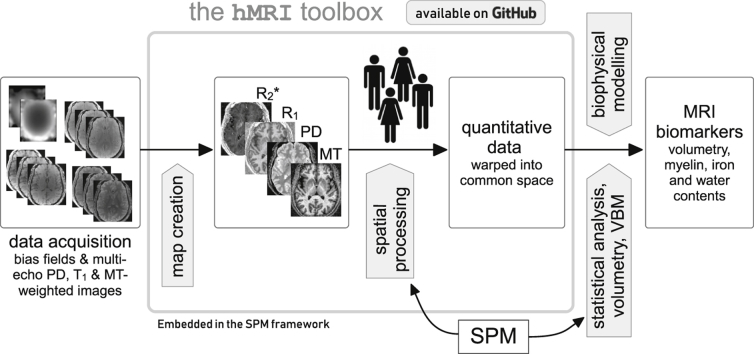

## 1 Introduction

Quantitative MRI (qMRI) finds increasing interest in neuroscience and clinical research because it is not only more sensitive, but also more specific, to microstructural properties of brain tissue such as axon, myelin, iron and water concentration than conventional weighted MRI (Cercignani et al., 2018; Assaf and Basser, 2005; Draganski et al., 2011; Lorio et al., 2014, 2016a; Stüber et al., 2014; Callaghan et al., 2015a). In conventional weighted MRI, the image grayscale values have arbitrary units and the value in a given voxel will depend on a large number of factors, such as the sequence type (e.g. the magnetisation-prepared rapid gradient echo, MPRAGE (Mugler and Brookeman, 1990) *versus* modified driven equilibrium Fourier transform, MDEFT (Deichmann et al., 2004) for T1-weighted anatomical images), sequence parameters (e.g. repetition time, TR, echo time, TE, or flip angle), and hardware effects (e.g. transmit and receive profiles and any scaling factors). In addition, the value will depend on multiple physical tissue properties such as the longitudinal and transverse relaxation times, $T_{1}$ and $T_{2}$, or the proton density, PD (Helms et al., 2009, 2010). qMRI accounts for these varied effects in order to increase the specificity of the estimated metrics and eventually quantify specific physical tissue properties (Cercignani et al., 2018; Lutti et al., 2010; Weiskopf et al., 2013). In qMRI, the estimated physical value has a direct meaning and is quantified in standardised units (e.g. $T_{1}$ in seconds) (Koenig et al., 1993). This standardised nature further increases the comparability across sites and time points (Deoni et al., 2008; Weiskopf et al., 2013), which may improve the sensitivity of multi-site studies and longitudinal analyses of development, plasticity and disease progression. A biophysical interpretation of physical qMRI parameters (Callaghan et al., 2015a; Stikov et al., 2015) or a combination of qMRI with biophysical modelling (e.g. Henkelman et al. (2001), Assaf and Basser (2005), or Mohammadi et al. (2015)) enables the *in vivo* characterisation of key microscopic brain tissue parameters, which previously could only be achieved with *ex vivo* histology. This concept is called *in vivo* histology using MRI (hMRI, Weiskopf et al. (2015)).

The estimation of quantitative and semi-quantitative metrics commonly includes one or more of the effective transverse relaxation rate ($R_{2}^{⋆}=1/T_{2}^{⋆}$), the longitudinal relaxation rate ($R_{1}=1/T_{1}$), the proton density (PD), the magnetisation transfer (MT) saturation and a number of diffusion MRI (dMRI) metrics (Draganski et al., 2011). However, the majority of fundamental and clinical neuroscience studies either refrain from acquiring qMRI data or the quantitative approach is limited to dMRI only. One reason for this might be that standardised qMRI imaging protocols (such as the protocol for the Human Connectome Project (Sotiropoulos et al., 2013)) and processing software (see a summary in Soares et al. (2013)) are readily available for dMRI but less so for other qMRI techniques.

Consequently, the neuroscience and clinical research community lacks a standardised qMRI implementation to handle the wide diversity of data acquisition types and estimate parameters such as $R_{2}^{⋆}$, $R_{1}$, PD and MT saturation, which are sensitive to iron, myelin, and water content in tissue microstructure, and thus provide complementary information to the axonal properties revealed by dMRI. For example, $R_{2}^{⋆}$ is often estimated using gradient recalled echo data acquired with multiple echo times (TE) to create high resolution maps that show strong contrast between different types of brain tissue (Bernstein et al., 2004). Similarly, PD and $R_{1}$ can be derived from two acquisitions varying the excitation flip angle (Wang et al., 1987; Deoni et al., 2005; Deoni, 2007; Schabel and Morrell, 2008; Helms et al., 2008a, 2011; Liberman et al., 2013; Heule et al., 2015; Baudrexel et al., 2016). For accurate estimation of the latter qMRI parameters, an implementation must adequately correct for instrumental biases such as inhomogeneous transmit (Lutti et al., 2010) and receive fields (Volz et al., 2012; Mezer et al., 2016; Lorio et al., 2018). Such correction should be based on the additional mapping of these fields, but the implementation should also provide solutions when these fields have not been measured. For example in clinical settings, MR sequences for estimating instrumental biases are often unavailable due to time, hardware or software constraints. For such studies, the facility to correct for instrumental biases retrospectively using image processing methods that do not rely on additional MRI acquisitions is highly desirable.

A number of open-source tools have been developed to support qMRI use and make qMRI more broadly accessible to neuroscience and clinical research (see for example qMRLab (Cabana et al. (2015), https://github.com/qMRLab/qMRLab), QUIT (Wood (2018), https://github.com/spinicist/QUIT), mrQ (Mezer et al. (2016), https://github.com/mezera/mrQ) and QMAP (https://www.medphysics.wisc.edu/∼samsonov/qmap/). These tools include, to various extents, data acquisition guidelines, tools for protocol simulation and optimisation, a multitude of models and methods for data fitting and estimation of qMRI parameters, and visualisation tools.

While most of these tools focus on model fitting and generation of qMRI maps, the question of spatial and statistical processing of these qMRI maps is not directly addressed. For spatial processing and group level statistical analysis, the established tools are primarily designed for diffusion MRI (see for example TBSS in FSL (Smith et al., 2006) and TRACULA (Yendiki et al., 2011) in FreeSurfer). In addition, a number of custom made tools have been developed based on established neuroimaging software, e.g.: applications of the FreeSurfer surface projection software (Dale and Sereno, 1993; Fischl et al., 1999; Fischl and Dale, 2000) to compare quantitative relaxation and susceptibility data on the cortex (Marques et al., 2017), or usage of the VBM framework (Ashburner and Friston, 2000) to process qMRI data across the whole brain on a voxel-by-voxel basis (Büchel et al. (2004) and Mohammadi et al. (2012) for dMRI metrics, and Table 1 for $R_{2}^{⋆}$, $R_{1}$, PD and MT saturation). One challenge common to all these tools is to find a proper method to locally preserve the qMRI parameters after (non-linear) spatial registration. The methods using the statistical parametric mapping (SPM) framework typically reduce residual misalignment between images by isotropic spatial smoothing. However, applying this framework directly to qMRI data would introduce partial volume effects at tissue boundaries and corrupt the quantitative values. Hence a modified smoothing approach, which aims to achieve within class smoothing only, is preferred. The majority of the studies in Table 1 took advantage of voxel-based quantification (VBQ), an approach introduced by Draganski et al. (2011) and developed for the comprehensive multi-parameter mapping (MPM) approach (Helms et al., 2008b, 2009; Weiskopf et al., 2011, 2013) to correct for potential error introduced by spatial smoothing.

**Table 1:** Review of studies related to the MPM acquisition protocol using predecessors of the hMRI-toolbox to make inference on myelin (My), iron (Fe), or the volume (Vol) measured by voxel-based morphometry (VBM). Note that all studies used Siemens MRI scanners. Abbreviations: $R_{1}=$longitudinal relaxation rate, $R_{2}^{\left(⋆\right)}=$ (effective) transverse relaxation rate, $PD^{\left(*\right)}=$ (effective) proton density, MT= magnetisation transfer saturation, C = controls, P = patients, dMRI= diffusion MRI, fMRI= functional MRI, MEG= magnetoencephalography, EEG= electroencephalography.

Reference	Predominant tissue feature			qMRI	Subjects	Remarks
	My	Fe	Vol	parameters		
Studies demonstrating improved volumetry						
Helms et al. (2009)	_	_	✓	MT	49 C	Improved segmentation of deep brain grey matter structures using magnetisation transfer saturation (MT) parameter maps.
Lambert et al. (2013)	_	_	✓	$R_{1}$, $R_{2}^{⋆}$, MT, $PD^{⋆}$	34 C	Brainstem segmentation using a modified multivariate mixture of Gaussians based on MPMs.
Studies of myelin and/or iron						
Draganski et al. (2011)	✓	✓	✓	$R_{1}$, $R_{2}^{⋆}$, MT	26 C	A fingerprint of age-dependent brain atrophy and underlying microstructural changes in myelin, iron deposits and water.
Lambert et al. (2013)	✓	✓	✓	$R_{1}$, $R_{2}^{⋆}$, MT, $PD^{⋆}$	26 C	Characterizing aging in the human brainstem using quantitative multimodal MRI analysis.
Freund et al. (2013)	✓	_	✓	$R_{1}$, MT	13 P/18 C	Atrophic and microstructural changes of corticospinal axons and sensorimotor cortical areas observed in the first months after spinal cord injury.
Callaghan et al. (2014)	✓	✓	✓	$R_{1}$, $R_{2}^{⋆}$, MT, $PD^{⋆}$	138 C	A whole-brain pattern of age-associated microstructural differences in the asymptomatic population providing insight into the neurobiology of aging.
Lorio et al. (2014)	✓	✓	✓	$R_{1}$, $R_{2}^{⋆}$, MT, $PD^{⋆}$	96 C	Deviation of volume changes assessed with VBM using standard T1-weighted or MT maps, attributed to age-related iron changes.
Lorio et al. (2016b)	✓	✓	✓	$R_{1}$, $R_{2}^{⋆}$, MT, $PD^{⋆}$	120 C	Impact of microstructural properties of brain tissue-myelination, iron, and water content on automated measures of brain morphology using VBM.
Steiger et al. (2016)	✓	✓	_	$R_{2}^{⋆}$, MT	31 C	Age-related increase of iron correlated negatively with iron and myelin in the ventral striatum, which predicted individual memory performance.
Whitaker et al. (2016)	✓	_	✓	$R_{1}$, MT	297 C	Assessment of layer-specific microstructure in adolescence development and comparison to volume differences.
Carey et al. (2018)	✓	✓	_	$R_{2}^{⋆}$, MT, $R_{1}$, $PD^{⋆}$	93 C	Delineation of cortical myelin profiles across cortical depths and mapping of tissue water sensitive parameters ($PD^{⋆}$).
Ziegler et al. (2018)	✓	✓	✓	$R_{2}^{⋆}$, MT	15 P/18 C	Trauma-induced neuroplastic processes in brain and spinal cord including neurodegenerative processes associated with iron and myelin changes.
Studies linking structure and function in the cortex						
Dick et al. (2012)	✓	_	_	$R_{1}$ (& fMRI)	9 C	Combining structural $R_{1}$ mapping with functional tonotopy to reveal their interrelation within the primary auditory areas (A1 and R).
Sereno et al. (2013)	✓	_	_	$R_{1}$ (& fMRI)	6 C	Combined structural $R_{1}$ mapping and functional retinotopy revealed boundaries visible in the $R_{1}$ maps that correspond to recognised retinotopic borders.
Lutti et al. (2014)	✓	_	_	$R_{1}$ (& fMRI)	–	Review about using high-resolution mapping of $R_{1}$ as an index of cortical myelination.
Helbling et al. (2015)	✓	_	_	$R_{1}$ (& MEG)	5 C	Combining non-invasive electrophysiology with MPMs demonstrating microstructure-function relationship.
Carey et al. (2017)	✓	_	_	$R_{1}$ (& fMRI)	10 C	Functional and $R_{1}$ mapping demonstrate overlap between articulator and R1 myelin proxy maps in pre and post-central regions.
Dick et al. (2017)	✓	_	_	$R_{1}$ (& fMRI)	8 C	Functional and $R_{1}$ mapping demonstrate strong concordance in the degree of cortical myelination and the strength of tonotopic activation across several auditory cortical regions.
Studies combining MPMs and biophysical models						
Callaghan et al. (2015a)	✓	✓	_	$R_{2}^{⋆}$, MT, $R_{1}$	138 C	Combining MPM and a general linear relaxometry model to describe the biophysical relation of $R_{1}$ on iron, myelin, and water content.
Mohammadi et al. (2015)	_	✓	_	MT (& dMRI)	38 C	Using MPM-based MT and diffusion-MRI-based tract fibre density as proxies for estimating myelin and axon densities as well as the g-ratio.
Weiskopf et al. (2015)	✓	✓	✓	$R_{2}^{⋆}$, MT, $R_{1}$, $PD^{⋆}$	–	Review about *in vivo* histology using MRI.
Callaghan et al. (2016b)	✓	✓	✓	$R_{2}^{⋆}$, MT, $R_{1}$, $PD^{⋆}$	12 C/30 C	Using a biophysical relation between $R_{1}$, MT, and $R_{2}^{⋆}$ to generate synthetic quantitative maps.
Ellerbrock and Mohammadi (2018)	✓	✓	_	MT, PD (& dMRI)	22 C	Comparing different proxies for myelin and fibre volume fractions using MPMs and diffusion MRI.
Edwards et al. (2018)	✓	✓	_	$R_{1}$, $R_{2}^{\left(⋆\right)}$, MT, PD (& dMRI)	22 C	Review on cortical models for *in vivo* histology.

A particular instantiation of qMRI developed at 3T, the MPM approach, spans data acquisition, modelling and bias correction of three multi-echo spoiled gradient echo volumes to generate $R_{2}^{⋆}$, $R_{1}$, PD, as well as semi-quantitative MT saturation maps. This framework enables time-efficient whole brain mapping with high isotropic resolution of 800 *μ*m in 27 min (Callaghan et al., 2015b) or reduced MPM protocol (no MT saturation) at 1 mm isotropic resolution in 14 min at 3T (Papp et al., 2016) and has even enabled the acquisition of ultra-high-resolution quantitative maps with 400 *μ*m resolution at 7T (Trampel et al., 2017). This framework has been used in a variety of fundamental and clinical neuroscience studies (Table 1) focussing on: (a) improving the segmentation of deep grey matter structures, (b) evaluating the myelin and iron concentration in the brain and spinal cord, (c) linking structure and function in the cortex, and (d) using the MPM parameter maps as proxies for biophysical tissue models.

In this paper, we present the hMRI-toolbox, a comprehensive open-source toolbox that streamlines all the processing steps required to generate $R_{2}^{⋆}$, $R_{1}$, PD and MT saturation maps and provides appropriate spatial processing for group analyses. The flexible nature of the toolbox makes it applicable to a wide range of data types, from the full MPM protocol to subsets of it, including single contrast echo trains for mapping $R_{2}^{⋆}$ or variable flip angle data for mapping of $R_{1}$ and PD using multi-echo or even single-echo data. The toolbox is embedded in the SPM framework (http://www.fil.ion.ucl.ac.uk/spm/software/spm12/), profiting from the highly accurate spatial registration into a common space and the variety of established statistical inference schemes. The spatial processing part of the toolbox can be applied to any set of rotationally-invariant qMRI maps, including a number of diffusion MRI parameters and all common qMRI metrics.

## 2 Background

### 2.1 The MPM protocol

The MPM multi-echo protocol was introduced in Weiskopf and Helms (2008) and Weiskopf et al. (2013) for estimating the longitudinal relaxation rate $R_{1}$, the effective transverse relaxation rate $R_{2}^{⋆}$, the proton density PD and the magnetisation transfer MT and generalises a number of acquisition protocols. It typically involves acquiring six to eight images at different echo times (TE) for each of the PD-, T1-and MT-weighted acquisitions in an RF and gradient spoiled gradient echo sequence (referred to as T1w, PDw and MTw echoes, respectively). The hMRI-toolbox can flexibly deal with a large range of site- and study-specific acquisition schemes, from the full MPM protocol to subsets of it, including single contrast echo trains for mapping $R_{2}^{⋆}$ or variable flip angle data for mapping of $R_{1}$ and PD using multi-echo or single-echo data, comparable to e.g. DESPOT1 (Deoni et al., 2005). Example MPM acquisition protocols can be found at http://hmri.info.

### 2.2 Overview theory of MPM signal model

**Fig. 1:**
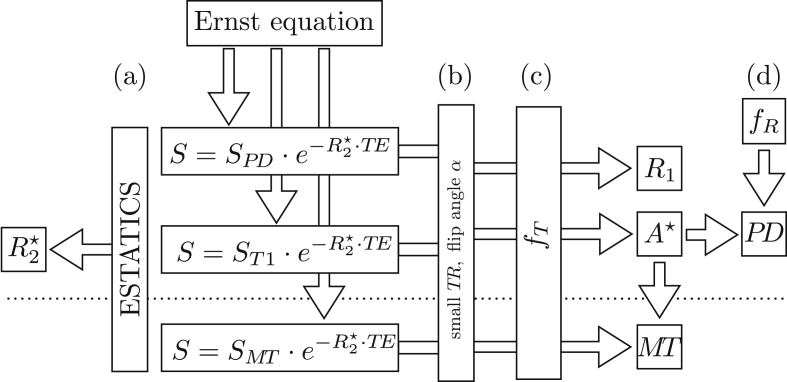
Overview of qMRI map generation from the weighted imaging and reference MPM data. The signal *S* is modelled by the Ernst equation with an exponential decay depending on the echo time TE. The longitudinal relaxation rate $R_{1}$, the effective transverse relaxation rate $R_{2}^{⋆}$, the proton density PD and the magnetisation transfer (MT) saturation are estimated from the data, using approximations for small repetition time TR and small flip angles *α*. The transmit and receive bias fields $f_{T}$ and $f_{R}$ are used to correct for instrumental biases.

We give here, and in Fig. 1, a short overview of the theory underlying the qMRI map creation process. For a more detailed outline of the theory and the estimation procedures applied in the hMRI-toolbox see Appendix A.

The signal from the multi-echo PDw, T1w and MTw acquisitions can be described by the Ernst equation (Ernst and Anderson, 1966; Helms et al., 2008a, b). The effective transverse relaxation rate $R_{2}^{⋆}$ can then be derived from the TE dependence of the signal. The unified description of the multi-echo data from all three contrasts into a single model, denoted as ESTATICS (Weiskopf et al., 2014), provides a more robust estimation of $R_{2}^{⋆}$ with a higher signal-to-noise ratio compared to separate estimations (Fig. 1a). Using approximations of the signal equations for small repetition time TR and small flip angles *α*, the longitudinal relaxation rate $R_{1}$, the apparent signal amplitude $A^{\text{*}}$ map (proportional to the proton density PD) and the magnetisation transfer MT can be estimated. At this point (Fig. 1b), the generated maps are biased by $B_{1}$ transmit $f_{T}$ (Fig. 1c) and receive $f_{R}$ (Fig. 1d) field inhomogeneities. The hMRI-toolbox provides correction methods for these bias fields based on specific $B_{1}$ transmit and receive field measurements or image processing methods. While $f_{T}$ influences the local flip angle and hence all three ($R_{1}$, PD, MT) maps are affected, the RF sensitivity bias field $f_{R}$ only influences the PD map (in the absence of subject motion).

The toolbox can also handle the situation where only a subset of data is available. For example, $R_{2}^{⋆}$, $R_{1}$ and PD can still be estimated when no MTw acquisitions are acquired, $R_{2}^{⋆}$ alone can be estimated when neither MTw nor T1w acquisitions are available (single multi-echo PDw data). $R_{1}$, PD and MT saturation maps can be generated from single echo PDw, T1w and MTw images, not requiring multi-echo acquisitions. The theory and map creation tools also encompass the creation of $R_{1}$ maps from other variable flip angle approaches, such as DESPOT1 (Deoni et al., 2005) or $R_{2}^{⋆}$ maps from multi-echo data, such as certain susceptibility mapping/weighted imaging approaches.

## 3 Methods

### 3.1 Toolbox documentation and installation

The latest version of the toolbox can be downloaded from the hMRI-toolbox page (http://hmri.info) as a zip file (containing the last official release) or by cloning the git repository (https://github.com/hMRI-group/hMRI-toolbox) to keep up-to-date with the latest incremental developments.

Updated documentation is available as a Wiki (https://github.com/hMRI-group/hMRI-toolbox/wiki). It includes installation instructions, an example dataset, a tutorial and a detailed description of the implemented functionalities. Information on releases and versioning, development and contribution guidelines are also provided.

The toolbox has been developed and tested with MATLAB versions 8.0 (R2012b) to 9.3 (R2017b) and SPM12 from version r6906 onwards. Since the hMRI developments will be synchronised with SPM developments, it is recommended to use the most recent SPM release (http://www.fil.ion.ucl.ac.uk/spm/software/spm12/) to benefit from the latest developments.

The hMRI-toolbox is free but copyright software, distributed under the terms of the GNU General Public License as published by the Free Software Foundation (as given in file Copyright.txt). Further details on “copyleft” can be found at http://www.gnu.org/copyleft/.

In particular, the hMRI-toolbox is supplied as is. No formal support or maintenance is provided or implied. Since the toolbox was developed for academic research, it comes with no warranty and is not intended for clinical use.

### 3.2 MPM example dataset

An MPM example dataset from a healthy subject for demonstrating the hMRI-toolbox features was acquired on a 3T Prisma system (Siemens Healthcare, Erlangen, Germany) at the Wellcome Centre for Human Neuroimaging, London, UK. This dataset can be downloaded from http://hmri.info and is described in detail in Callaghan et al. (submitted).

### 3.3 Organisation of the toolbox

**Fig. 2:**
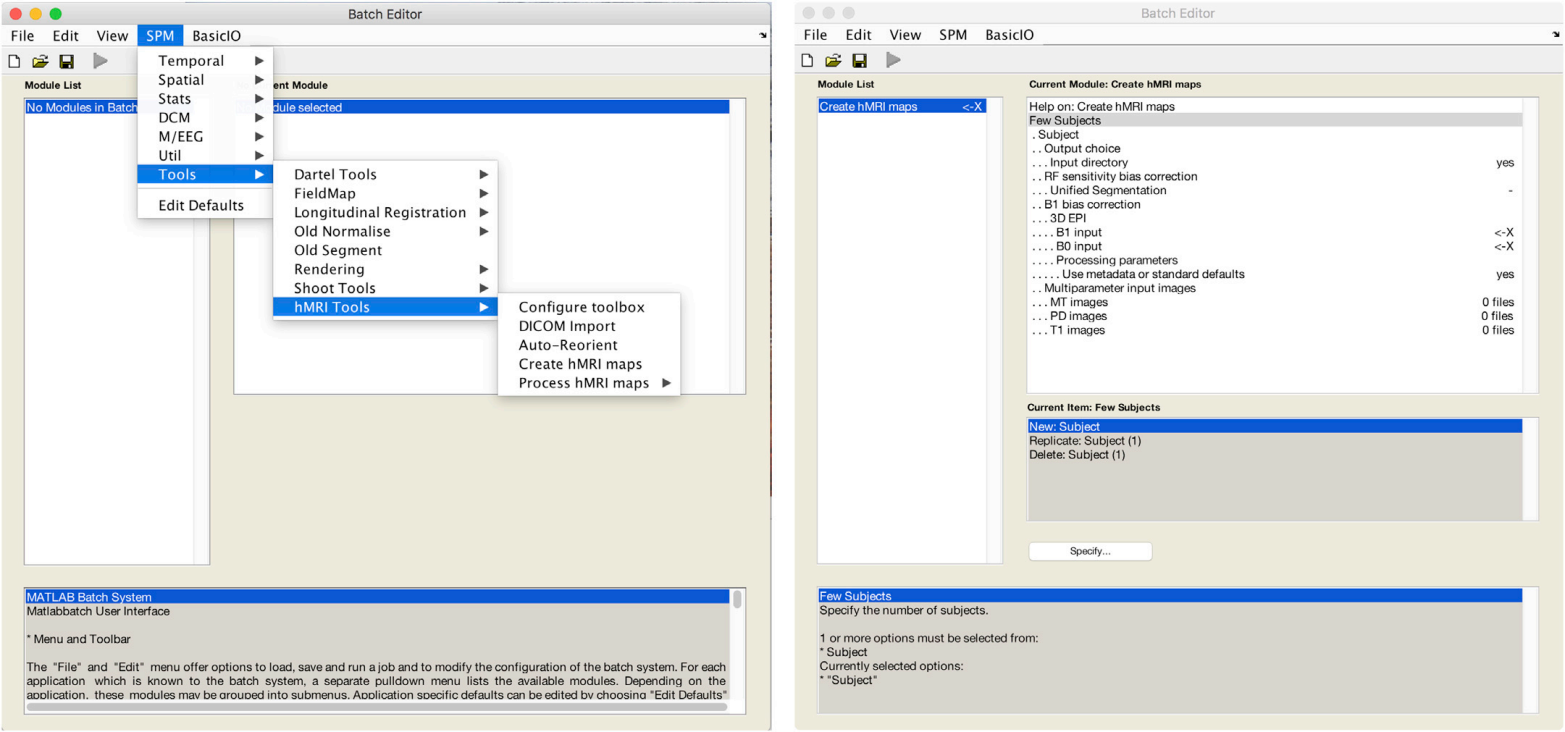
Left: After installation (Section 3.1), the hMRI toolbox can be started from the SPM menu of the batch editor. Five options include toolbox configuration, DICOM import, re-orientation of the data to MNI space, creation and processing of the hMRI maps. Right: Input mask for the map creation part of the toolbox.

The hMRI-toolbox is organised into five main modules (Fig. 2): *Configure toolbox*, *DICOM import*, *Auto-reorient*, *Create hMRI maps* and *Process hMRI maps*. While the *Configure Toolbox* and *Process hMRI maps* modules can be run for a group of subjects, the *DICOM Import*, *Auto-reorient* and *Create hMRI maps* modules must be run for each subject and each session separately (if several datasets acquired per subject, e.g. in a longitudinal study). A brief description of each module is provided below and details can be found in the Appendices.

### 3.4 *Configure toolbox* module

The hMRI-toolbox provides the user with a set of default acquisition and processing parameters for most common acquisition protocols without any further customisation. However, customisation is possible and necessary to broaden the toolbox usability to a wider range of protocols, scanners and vendors. Therefore, the *Configure toolbox* module allows the user to select either standard or customised default parameters, to match the user's own site- or protocol-specific setup and to be used in the subsequent modules (see details in Appendix C.1).

### 3.5 *DICOM import* module

*DICOM import* is a tool to convert DICOM data into NIfTI files. During conversion, the whole DICOM header is stored as JSON-encoded metadata in a file along side the NIfTI images. This feature is implemented in SPM12 from release r7219 (November 2017), following the hMRI-toolbox implementation. Note that Philips-specific rescaling factor (Chenevert et al., 2014) is applied at conversion, starting from version v0.2.0 of the hMRI-toolbox and release r7487 of SPM12. The hMRI-toolbox also provides metadata handling functionalities to retrieve parameter values and store processing parameters in the JSON-encoded metadata file (see Table 3 for an example of processing information stored as metadata). Detailed description of the DICOM import module, the metadata handling tools and BIDS compliance aspects (Gorgolewski et al., 2016) is provided in Appendix B.

### 3.6 *Auto-reorient* module

The reorientation of the images towards a standard pose, setting the anterior commissure at the origin and both anterior and posterior commissure (AC/PC) in the same axial plane, as defined in MNI space (Mazziotta et al., 1995, 2001a,b), is a common step that increases the consistency in individual head positions prior to normalisation or segmentation. For example, SPM's segmentation (Ashburner and Friston, 2005) is sensitive to the initial orientation of the images. Therefore, *Auto-reorient* provides a simple tool for automatically and uniformly reorienting a set of images prior to any further processing including multi-parameter map calculation. Note, that the reorientation modifies the orientation information in each image header, but the reoriented images are not resliced. For more details, see Appendix C.2.

### 3.7 *Create hMRI maps* module

The *Create hMRI maps* module computes quantitative as well as semi-quantitative estimates of $R_{2}^{⋆}$, $R_{1}$, PD and MT saturation from unprocessed multi-echo T1w, PDw and MTw spoiled gradient echo acquisitions. The map creation module corrects the qMRI estimates for spatial receive (Section 3.7.3) and transmit (Section 3.7.2) field inhomogeneities.

#### 3.7.1 Multi-parameter input images

**Fig. 3:**
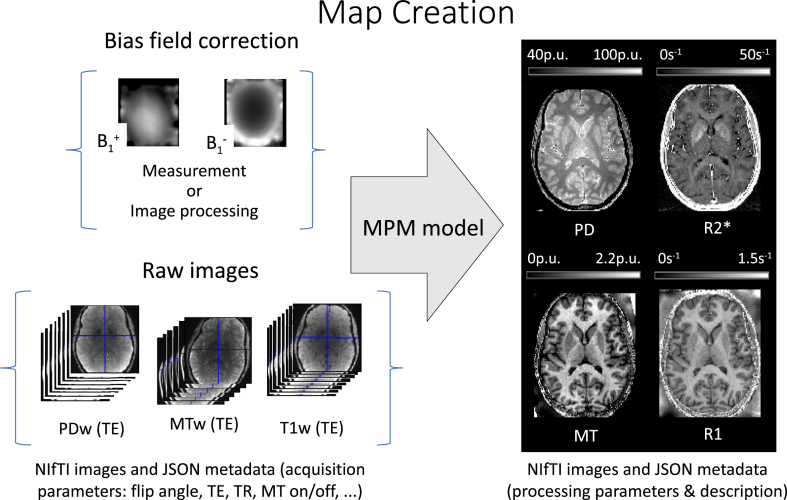
Map creation workflow illustrated for the MPM example dataset (Callaghan et al., submitted). The MPM protocol includes three multi-echo spoiled gradient echo scans with predominant T1-, PD- and MT-weighting achieved by an appropriate choice of the repetition time, the flip angle and the off-resonance MT pulse. Optional RF transmit ($B_{1}^{+}$) and receive ($B_{1}^{-}$) field measurements can be added to the protocol, improving the quality of instrumental bias correction in the MPM maps. Alternatively, these reference measurements can be, to a limited extent, replaced by dedicated image processing steps that are provided by the toolbox. The map creation module generates PD, $R_{1}$, MT and $R_{2}^{⋆}$ maps. For each map, a JSON metadata file is created, which contains information about the processing pipeline of each image (see example in Table 3).

The module takes the (possibly reoriented) series of multi-echo spoiled gradient echo images as input for the creation of quantitative as well as semi-quantitative maps (Fig. 3) of $R_{2}^{⋆}$, $R_{1}$, PD and MT saturation (Helms et al., 2008a, b; Weiskopf et al., 2013, 2014) as described in the Background Section 2 and Appendix A. The number and quality of the output maps depends on the contrasts (PDw, T1w, MTw) and number of echoes available, and on the availability of additional bias field measurements. A single multi-echo PDw contrast allows for the calculation of a single $R_{2}^{⋆}$ map. If T1w images are additionally provided, $R_{1}$ and PD maps will also be generated. The MT map estimation requires the acquisition of additional MTw images. The map creation also involves optional correction for $B_{1}^{+}$ ($B_{1}$ transmit) bias field $f_{T}$ (Section 3.7.2) and the $B_{1}^{-}$ (RF receive sensitivity) bias field $f_{R}$ (Section 3.7.3) as well as spoiling imperfections (Yarnykh, 2010; Preibisch and Deichmann, 2009).

#### 3.7.2 B_1_ (transmit) bias correction

The map creation module includes the determination of $B_{1}$ transmit bias field maps ($f_{T}$ expressed in p.u. Of the nominal flip angle) for transmit bias correction of the quantitative data. Several methods are implemented. Depending on the choice of the specific method the GUI requires the user to provide adequate input files. Further details on the supported correction methods can be found in Appendix C.3 and the respective original publications (Lutti et al., 2010, 2012; Weiskopf et al., 2013, 2011; Yarnykh, 2007; Chung et al., 2010).

#### 3.7.3 Receiver RF sensitivity bias correction

Three options are available to correct for RF receive sensitivity bias ($f_{R}$) within the *Create hMRI maps* module. Two of them rely on measured RF sensitivity maps (Single or Per contrast options) while the third method is data driven (Unified Segmentation option: no input sensitivity map required). Although not recommended, it is possible to disable RF sensitivity correction altogether by selecting the None option. While options Single and Unified Segmentation assume that the sensitivity profile is consistent between contrasts (i.e. small inter-contrast subject movement is assumed), the Per contrast option accounts for inter-contrast variation in RF sensitivity profile due to larger subject motion (Papp et al., 2016). Details on the different RF sensitivity bias correction methods can be found in Appendix C.4 and in the respective publications (Papp et al., 2016; Weiskopf et al., 2013).

#### 3.7.4 Output

By default, the estimated quantitative maps are output into a Results subdirectory within the folder of the first PDw echo. Alternatively, a user-defined folder for the output of the toolbox can be selected in which the Results directory will be created. The estimated qMRI maps are saved in the Results directory, with supplementary files are output in the Results/Supplementary subfolder. The basename for all qMRI maps is derived from the first echo of the PDw image series, see Table 2 for brief description. If data is reprocessed, a new sub-folder is created.

**Table 2:** Output files from the *Create hMRI maps* module using the SE/STE $B_{1}$ mapping and per-contrast RF sensitivity bias correction.

Results directory	Description
<firstPDfileName>_MTsat.[nii|json]	Estimated MT saturation map in p.u.
<firstPDfileName>_PD.[nii|json]	Estimated PD map in p.u.
<firstPDfileName>_R1.[nii|json]	Estimated $R_{1}$ map in $s^{-1}$
<firstPDfileName>_R2s_OLS.[nii|json]	Estimated $R_{2}^{⋆}$ map in $s^{-1}$ (ESTATICS) with ordinary least squares fit
Results/Supplementary directory	Description
hMRI_map_creation_rfsens_params.json	RF sensitivity bias correction parameters (measured sensitivity maps)
hMRI_map_creation_b1map_params.json	$B_{1}$ transmit map estimation: acquisition and processing parameters
hMRI_map_creation_job_create_maps.json	Create hMRI maps: acquisition and processing parameters
hMRI_map_creation_mpm_params.json	Acquisition and processing parameters used for the current job
hMRI_map_creation_quality_assessment.json	Quality assessment results (Appendix D)
<firstSESTEfileName>_B1map.[nii|json]	Estimated $B_{1}$ bias field $f_{T}$ map (p.u.)
<firstSESTEfileName>_B1ref.[nii|json]	Anatomical reference for $B_{1}$ bias field correction
<firstPDfileName>_MTw_OLSfit_TEzero.[nii|json]	MTw echoes extrapolated to TE=0
<firstPDfileName>_PDw_OLSfit_TEzero.[nii|json]	PDw echoes extrapolated to TE=0
<firstPDfileName>_R2s.[nii|json]	Estimated $R_{2}^{⋆}$ map from simple exponential fit (PDw echoes)
<firstPDfileName>_T1w_OLSfit_TEzero.[nii|json]	T1w echoes extrapolated to TE=0

### 3.8 *Process hMRI maps* module

**Fig. 4:**
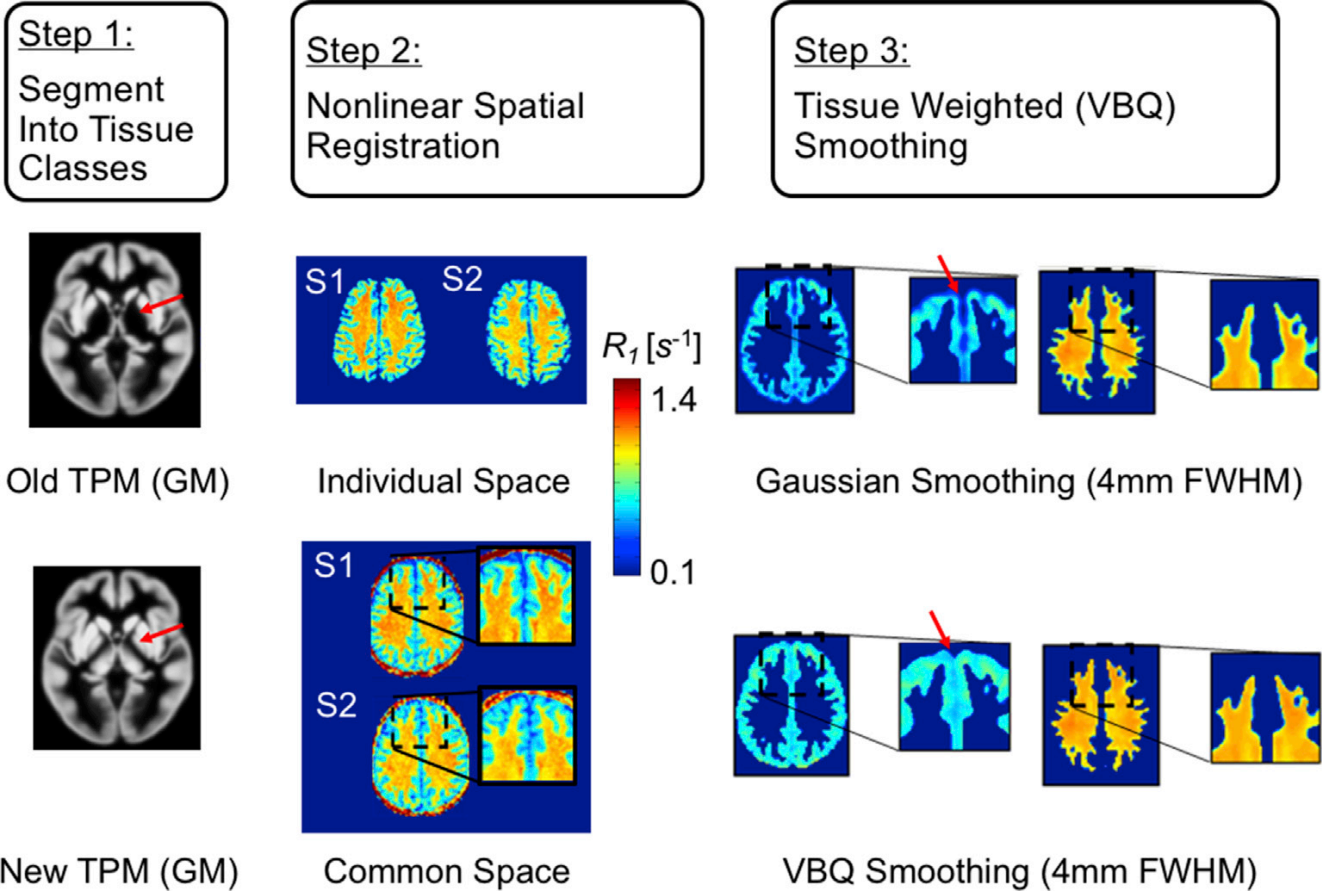
Overview of the spatial processing module. It consists of three steps: (1) segmentation, (2) highly parametrised non-linear spatial registration, and (3) tissue-weighted smoothing. The segmentation step (1) uses novel tissue-probability maps (TPMs), designed to take advantage of the better contrast of the MPMs for improved segmentation (exemplified for the deep-grey matter by the arrow in (1)). The non-linear spatial registration step into common space (2) reduces inter-individual anatomical differences (exemplified for $R_{1}$ maps of subject one and two, S1 and S2, respectively). To further reduce residual anatomical differences (see magnification boxes in (2)) and enhance statistical inference, the qMRI maps can be spatially smoothed (3) using the voxel-based quantification (VBQ) smoothing procedure. As compared to Gaussian smoothing, VBQ smoothing avoids bias in the qMRI maps (e.g. see arrows in (3), highlighting a rapid decline in $R_{1}$ values at tissue boundaries only after Gaussian smoothing. The VBQ smoothing is detailed in Eq. (C.3) and Draganski et al. (2011)). The sub-figure (1) has been adapted from (Lorio et al., 2016a), sub-figures (2) and (3) from Mohammadi and Callaghan (2018).

The *Process hMRI maps* module provides dedicated tools and tissue probability maps for the spatial processing of quantitative MRI maps based on the corresponding SPM framework. The spatial processing pipeline for hMRI data relies on three main operational steps (Fig. 4): (1) segmentation (Ashburner and Friston, 2005; Draganski et al., 2011; Lorio et al., 2016a), (2) non-linear spatial registration into common space (Ashburner (2007)) and (3) tissue-weighted smoothing (Draganski et al. (2011)), using three different sub-modules that are further detailed in the Appendix, Table 4. Furthermore, a fully integrated processing pipeline is provided as an additional sub-module to facilitate standard data processing without the need to combine the individual steps in this module. Details on the three sub-modules and the integrated pipeline are provided in Appendix C.5.

### 3.9 Statistical analysis

The standard SPM statistical analysis and modelling approaches such as mass-univariate General Linear Modelling can be applied to the spatially processed maps, see, e.g., Draganski et al. (2011) and Freund et al. (2013). Additionally, the multiple parameter maps lend themselves to multi-variate analyses approaches as well (Draganski et al., 2011).

## 4 Discussion and outlook

This paper introduced the hMRI-toolbox, which is embedded in the SPM framework and allows for the estimation and processing of four quantitative MRI parameter maps: the longitudinal and effective transverse relaxation rates $R_{1}$ and $R_{2}^{⋆}$, the proton density PD and the (semi-quantitative) magnetisation transfer saturation MT. This introduction includes a comprehensive summary of the MPM signal model as well as the currently available correction methods for the various bias sources that, if not corrected for, might impair the quantification. Finally, the processing steps for a dedicated SPM analysis of quantitative MRI maps (denoted as the VBQ approach) were presented, correcting for the potential partial volume effects introduced by spatial smoothing.

The name of the toolbox (h-MRI) originates from the concept of *in vivo histology* of tissue microstructure using MRI (Weiskopf et al., 2015). Hereby, the quantitative parameter maps generated with this toolbox provide key input parameters for biophysical models that are designed to non-invasively estimate specific microstructural tissue properties (see Table 1 for example studies).

*Considerations and interpretation of the MPM approach*. While the signal model (see Appendix A for details) used in this toolbox is based on the Ernst equation and thus provides a comprehensive means of calculating a set of physical quantitative (and semi-quantitative) parameters, including PD, $R_{2}^{⋆}$, $R_{1}$ and MT, we would like to emphasise that more sophisticated models can be derived to relate the parameters more directly to the underlying biophysical mechanisms and tissue characteristics.

The contrast provided by the $R_{2}^{⋆}$ metric is associated with different realisation of iron deposits (for a review, see e.g. Edwards et al. (2018)), myelination of axons (Marques et al., 2017), their orientation (Oh et al., 2013) and chemical exchange (Does, 2018). Multi-compartment models can be used, e.g., to separately describe the orientation-dependence of myelinated fibre pathways in $R_{2}^{⋆}$ parameter maps (e.g., Lee et al. (2016); Alonso-Ortiz et al. (2018); Wharton and Bowtell (2012)).

At a given field strength, the $R_{1}$ contrast is determined by the micro-structural tissue properties such as the local mobility of water molecules, the macromolecular content and the local concentration of paramagnetic ions such as iron or gadolinium-based contrast agents. It has been shown that $R_{1}$ depends within limits linearly on these tissue properties (Fatouros and Marmarou, 1999; Fatouros et al., 1991; Kaneoke et al., 1987; Shuter et al., 1998; Donahue et al., 1994; Kamman et al., 1988; Fullerton et al., 1982; Gelman et al., 2001). The $R_{2}^{⋆}$ and MT metrics can be used to describe the dependence of $R_{1}$ on these components (Callaghan et al., 2015a).

MT saturation is a proxy measure of the bound-pool water fraction (Helms et al., 2008b). It provides information about the macromolecular content of the micro-structural environment and is often used as a marker for myelin content, see e.g. Freund et al. (2013) and Callaghan et al. (2014). Under equivalent conditions for the off-resonance pre-pulse the same MT saturation values are expected. However, this will not be the case if the properties of the pre-pulse are changed across measurements. Therefore, we refer to the MT saturation measure as being semi-quantitative. The MT saturation map differs from the commonly used MT ratio (MTR; percent reduction in steady state signal) by explicitly removing the bias introduced by the spatially varying $T_{1}$ relaxation time and $B_{1}$-transmit field (Helms et al., 2008b). Additional minor corrections for $B_{1}$ transmit field inhomogeneity in the MT maps were applied as described in Weiskopf et al. (2013). The reduced spatially varying bias leads, e.g., to a higher contrast in deep brain structures than MTR and to reduced variance in the data (Callaghan et al., 2016b). Note that the MT saturation measure does not only depend on the bound-pool fraction but also on the exchange between the bound and free water pools (see e.g. Battiston and Cercignani (2018)). A more direct measure of the bound-pool fraction is provided by quantitative MT (qMT), which requires more time-consuming data acquisition, typically limiting the qMT map's spatial resolution (e.g., 2 mm isotropic in Stikov et al. (2011)).

*Sources of bias in qMRI and limitations to bias corrections*. Correction of instrumental characteristics and artefacts is an essential prerequisite for quantitative MRI. Sources of biases and artefacts include primarily transmit and receive fields, imperfect spoiling, $T_{2}^{⋆}$-bias and head motion. The artefact correction methods provided in the hMRI-toolbox are highly flexible, offering solutions to process reference measurements (e.g. transmit/receive field measurements carried out with a number of customised or product sequences) to correct for instrumental artefacts, as well as achieve optimal results even when no adequate measurements are available.

**Fig. 5:**
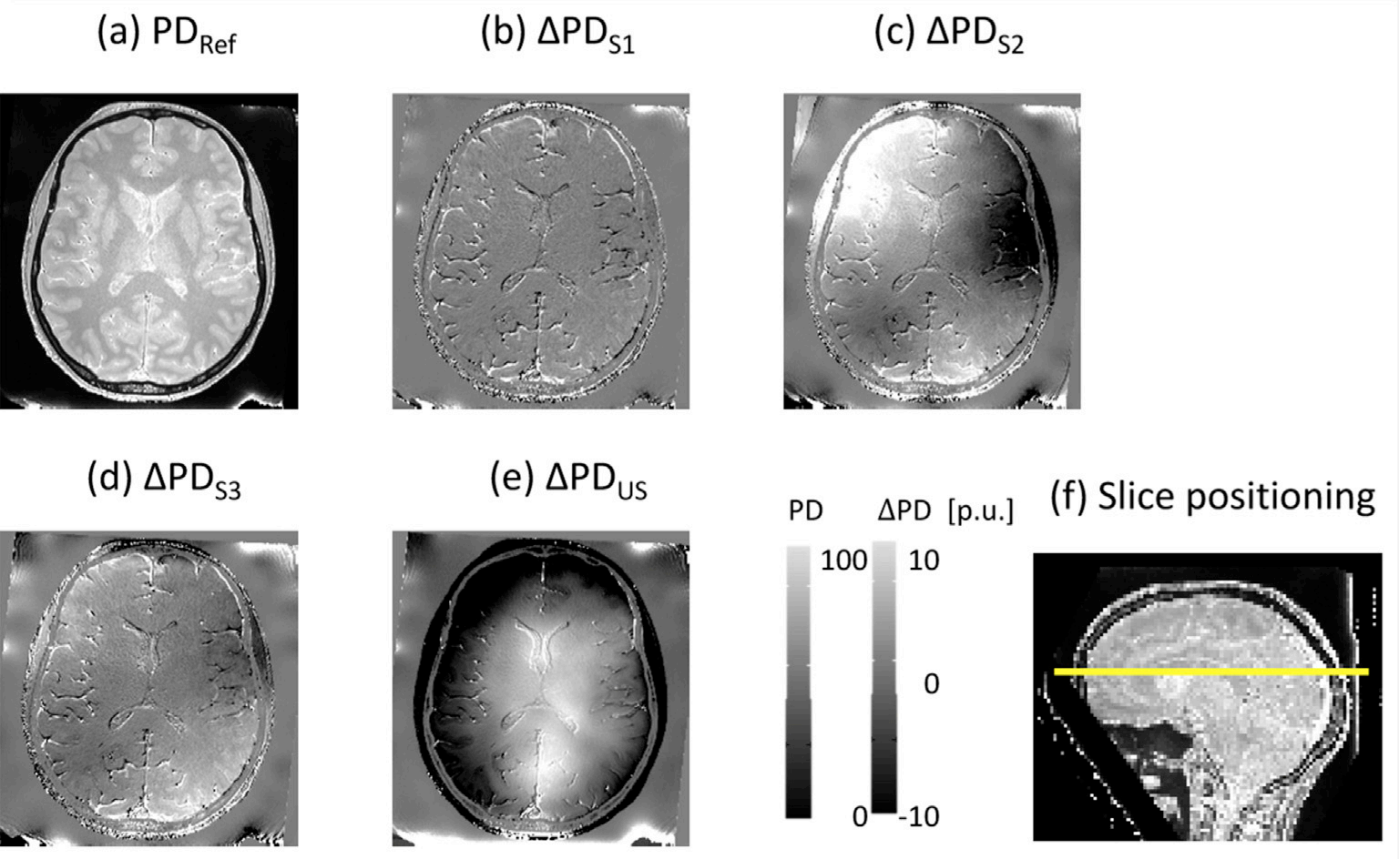
Comparison of different available RF sensitivity bias field correction methods (Appendix C.4) demonstrated on the MPM example dataset (Section 3.2) and PD difference maps. (a) PD map calculated using the reference correction method ($PD_{Ref}$), following the approach in Papp et al. (2016), requiring three sensitivity maps, each acquired directly before the respective PDw, MTw and T1w contrasts. (b–d) Difference between $PD_{Ref}$ and PD maps calculated from PDw and T1w images corrected by a single sensitivity map, acquired directly before the PDw images (b: $\text{Δ}PD_{S1}$), the MTw images (c: $\text{Δ}PD_{S2}$) and the T1w images (d: $\text{Δ}PD_{S3}$), respectively. Due to the large overt movement preceding the acquisition of the MTw images and corresponding RF sensitivity measurement (see Callaghan et al. (submitted)), there is a large discrepancy between head position for that specific RF sensitivity measurement on the one hand and head positions for the PDw and T1w images used to generate the PD map on the other hand. As a result, errors in (c) are much larger than in (b) and (d). (e) Difference between $PD_{Ref}$ and the PD map corrected for RF sensitivity bias using the Unified Segmentation approach ($\text{Δ}PD_{US}$). The body coil sensitivity profile, not corrected for in the reference method, modulates the PD difference map in (e). (f) Sagittal view of PD map in (a) depicting the position of the slice shown in (a–e).

**Fig. 6:**
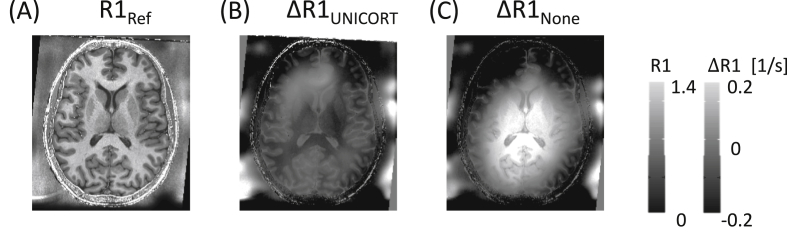
Comparison of two different available transmit field correction methods demonstrated on longitudinal relaxation rate ($R_{1}$) maps. (A) The $R_{1,Ref}$ map is depicted after using the reference correction method, following the $B_{1}$ mapping approach by Lutti et al. (2012). (B) The difference between the reference map $R_{1,Ref}$ and the $R_{1}$ map derived with UNICORT $B_{1}$ transmit bias correction (image processing method, no additional measurement required, see Weiskopf et al. (2011) for details). (C) The difference between the reference map $R_{1,Ref}$ and the $R_{1}$ map derived without $B_{1}$ transmit bias correction. (B) Shows a comparatively small residual modulation across the slice while (C) is strongly biased by the $B_{1}$ transmit inhomogeneity across the slice. The slice location in (A–C) is depicted in Fig. 5f.

The ideal imaging protocol includes dedicated measurements of transmit and receive field inhomogeneities, which are typically based on customised sequences (Fig. 5a–d for RF receive bias correction, and Fig. 6a for $B_{1}$ transmit bias correction). When customised sequences are not available, a standard spoiled gradient echo product sequence can be used to acquire data for low-resolution receive field mapping. Similarly, when not available, customised reference and calibration sequences such as 3D_EPI (Lutti et al., 2010, 2012) or 3D_AFI (Yarnykh, 2007) for correction of $B_{1}$ transmit effects can be replaced by manufacturer's service sequences such as TFL_B1_map (Chung et al., 2010) or RF_map (as examples on Siemens scanners). The toolbox provides the option to process data from several different $B_{1}$ transmit bias field mapping techniques or to use $B_{1}$ transmit bias field maps pre-computed outside the toolbox (see section 3.7.2 and Appendix C.3).

The measured transmit and receive fields can be affected by diverse sources of error leading to imperfect corrections. For example, residual misalignment between measured receive (or transmit) field and the spoiled gradient echo images can be one reason for such imperfections. In particular, when between-contrast (PDw, T1w, MTw) motion is large, discrepancies between head position for a single receive (or, to a lesser extent, transmit) field measurement and head position for all or some of the spoiled gradient echo images lead to additional motion-related bias in the quantitative maps (Fig. 5). In such a case, a per-contrast RF receive sensitivity measurement is preferable and can account for the between-contrast dynamic variation (see Appendix C.4, *Receive field sensitivity measurements* and Fig. 5). However, measured RF receive sensitivity maps such as described in Appendix C.4 can also suffer from residual modulations by the receive field of the body coil, which serves as a reference and whose inhomogeneity is not accounted for. Such modulation cannot be directly corrected for using the measured transmit field of the body coil at 3T and higher fields due to the non trivial applicability of the reciprocity principle at such field strengths (Hoult, 2000). As a result, when no large between-contrast motion is observed, RF sensitivity bias correction using the data driven receive field estimation (described in Appendix C.4) may prove more effective altogether. Such a data driven method could also be applied (with specific optimisation of the US regularisation parameters to the body coil's receive field profile) to correct for the above residual body coil receive field modulation. Finally, at 7T where RF body coils are not available, the currently implemented RF sensitivity measurements and bias correction approach are not applicable. All the above aspects are active fields of investigation, optimisation and validation that are also an integral part of the future developments of the toolbox. Similarly, $B_{1}$ transmit field mapping techniques can be inaccurate. For a comparison of the frequently used $B_{1}$-transmit field mapping techniques and a description of their respective accuracy and sources of uncertainty we refer to Lutti et al. (2010) and Pohmann and Scheffler (2013).

When no transmit and/or receive field inhomogeneity maps have been measured, which often happens in clinical settings due to time constraints, the toolbox provides the option to use image processing methods based on the Unified Segmentation approach (Ashburner and Friston, 2005) for $B_{1}$ transmit bias correction (UNICORT, Weiskopf et al., 2011; see Fig. 6b) or RF sensitivity bias correction (Fig. 5e). The Unified Segmentation approach takes advantage of the fact that bias corrections can be applied post hoc in good approximation for small read-out flip angles and short TR (Helms et al., 2008a). This requires no additional acquisition time but produces quantitative maps of lesser accuracy with some residual receive and/or transmit field modulation (Weiskopf et al., 2011; Baudrexel et al., 2016) compared to a correction with measured references. Note that the Unified Segmentation approach, whether applied for $B_{1}$ transmit (UNICORT) or RF sensitivity bias correction, has been optimised for the Siemens TIM-TRIO MR system using the body RF coil for transmission and the 32-channel receive head coil (Weiskopf et al., 2011). The corrections will perform appropriately for coils with similar transmit or receive field profiles, but might require further adjustments otherwise (see Weiskopf et al. (2011) for UNICORT optimisation). For more details, see (Callaghan et al., 2016a) and the hMRI wiki (the latter also provides further information on customized usage).

The proposed MPM protocol uses RF and gradient spoiling to minimise undesired transverse net magnetisation. Imperfect spoiling, which depends on the precise sequence protocol settings, can leave a residual bias in the $R_{1}$ map if no further correction is used (Preibisch and Deichmann, 2009; Yarnykh, 2010). For specific MPM protocols using the customised sequences, the hMRI-toolbox provides a correction for imperfect spoiling, see Eq. (A.15) in Appendix A.5. By default this correction is disabled but can be enabled through the toolbox customisation provided by the *Configure toolbox* module (Appendix C.1 and the hMRI-toolbox Wiki).

The estimation of PD maps can be biased by $T_{2}^{⋆}$ relaxation effects if not accounted for (Eq. (A.4) in Appendix A). Two correction methods, based on extrapolation of the data to TE=0 (Ellerbrock and Mohammadi, 2018) or relying on the estimated $R_{2}^{⋆}$ maps respectively, are implemented in the toolbox (Balteau et al., 2018). These correction methods require a number of echoes to be acquired for a robust fit of the exponential decay to derive TE=0 magnitude images and $R_{2}^{⋆}$ maps. In the case of a single-echo variable flip angle dataset, the $T_{2}^{⋆}$-weighting correction cannot be applied and the estimated PD and (to a much lesser extent) the $R_{1}$ and MT maps will be biased by $T_{2}^{⋆}$-modulations. Also, $R_{2}^{⋆}$ maps are biased in areas with severe susceptibility artifacts (Yablonskiy, 1998). Note that the JSON metadata file associated with the respective PD parameter map contains information about the processing steps and thus of potential $T_{2}^{⋆}$ modulation.

Head motion is widely recognised as a major source of artefacts in MR images, with severe consequences for quantitative MRI and morphological measures of the brain (Callaghan et al., 2015b; Weiskopf et al., 2014; Reuter et al., 2015). While quantitative measures of image quality have been introduced, visual inspection remains the most common means of rating data quality despite its limited sensitivity and inter-rater variability (Rosen et al., 2018). The hMRI-toolbox provides summary measures of head motion within and between the acquisitions of each image volume (intra- and inter-scan motion) (Castella et al. (2018) and Appendix D). The provided index of intra-scan motion has been tested against the history of head motion, recorded in real-time during the scans (Castella et al., 2018). Note that these intra- and inter-scan head motion measures could potentially be combined to guide toolbox users to objectively classify their data according to quality, for example to exclude or downweight poor-quality data of individuals in a statistical group analysis.

*Spatial processing pitfalls*. Since spatial processing in the hMRI-toolbox is embedded in the SPM framework, it is subject to the same limitations as any typical VBM study, including spatial normalisation accuracy, segmentation errors and partial volume effects (Ashburner and Friston, 2000; Ridgway et al., 2008; Focke et al., 2011). Auto-reorient is an option that can help improve the segmentation. However, it has to be done carefully. Poor signal-to-noise ratio, contrast-to-noise ratio, or outliers in the MPM input images may impair the reorientation procedure. In general, it is good practice to visually inspect the results of the hMRI pipeline to detect any obviously suspicious results. Piloting the processing pipeline using a batch for a single healthy subject dataset, combined with a careful check of the log files (in Results/Supplementary, metadata including summary description as shown in Table 3), and comparison with the computed MPM maps from the sample dataset, is recommended.

Residual misalignments between qMRI maps of individual participants in common space will be present despite the high degree of spatial correspondence that can be achieved by the non-linear warping algorithms available in SPM, e.g., by DARTEL (Ashburner, 2007). These misalignments are typically reduced in the VBM-framework by spatial smoothing. To correct for the partial volume effects at tissue boundaries that can be introduced by spatial smoothing, the hMRI-toolbox provides the dedicated smoothing approach that has been described in Fig. 4.

The choice of the appropriate smoothing kernel and its performance compared to alternative methods (e.g. TBSS (Smith et al., 2006) or TSPOON (Lee et al., 2009)) is still a subject of active research. An alternative method for reducing spatial misregistration in the cortex might be surface-based registration algorithms (Davatzikos and Bryan, 1996; Drury et al., 1996; Thompson and Toga, 1996; Fischl et al., 1999). However, a comparison study by Klein et al. (2010) between volume-based and surface-based registration methods could not demonstrate clear superiority of one or the other approach. In fact this is an active area of research to better understand the relative benefits and pitfalls of each approach (Canna et al., 2018).

*hMRI-toolbox for different MRI scanner platforms*. The first task for interested users of the hMRI-toolbox is the setup of the MPM acquisition protocol. To facilitate standardisation, a set of example protocols for the customised MPM sequences on Siemens platforms as well as standard sequences available on Siemens and Philips platforms are provided on the http://hmri.info website. Those protocols take advantage from the fact that MPM sequences primarily rely on multi-echo spoiled gradient echo sequences that are available on all modern MRI scanners. Even though the experience with implementing the specific MPM protocol on the Philips platforms is limited, first important steps have been achieved (Lee et al., 2017, 2018), and the MPM framework together with the hMRI-toolbox will be used in a multi-site clinical trial (NISCI trial, (Seif et al., 2018),) including Philips and Siemens MR systems.

Multi-scanner and multi-vendor data sets will require adjustments in terms of data handling and processing with the hMRI-toolbox. Most MPM studies up to now were carried out on Siemens MRI scanners using customised MPM sequences. Consequently, the toolbox is optimised for this scenario. New issues might arise when implementing the MPM protocol and using data from other vendors or conventional product sequences. For instance, different MT pre-pulse implementations will lead to changes of the MT saturation map, which will require appropriate inter-scanner calibration (Volz et al., 2010; Seif et al., 2018). Moreover, not every DICOM to NIfTI conversion software appropriately handles image intensity scaling, as reported e.g. for Philips data (Chenevert et al., 2014), leading to spurious intensity differences affecting the quantification. Making the hMRI-toolbox data formats fully BIDS compliant (Gorgolewski et al., 2016) by defining the ontology of acquisition parameters necessary for the creation of the quantitative maps (see Appendix B.5) further supports the use on multiple platforms and vendors, and is a high priority of the ongoing developments.

Applicability of the toolbox to different MRI platforms also involves ultra-high field MR systems. With the fast progress of ultra-high field scanners (7T and higher) on the MRI market, high-resolution data will become more routinely available and provide access to e.g. laminar-specific information (see 400 *μ*m new generation MPM, Trampel et al. (2017)), while also posing new challenges (e.g. $B_{1}^{+}$ field inhomogeneities) that the hMRI-toolbox will have to address.

*Future directions*. The hMRI-toolbox has been developed as a scientific collaborative project. As such its developments aim at making it broadly available, capitalising on its flexible and open-source implementation, and adjusting to data sets acquired on multiple MRI platforms (see *hMRI-toolbox for different MRI scanner platforms* above).

Sensitivity to small inter-individual changes of microstructure (e.g. plasticity) and the variation of change across subjects (e.g. in development, see https://www.biorxiv.org/content/early/2018/07/26/328146) is another challenge for longitudinal qMRI. To that end, the bias of the qMRI estimates in the presence of motion has been investigated (Weiskopf et al., 2014; Callaghan et al., 2015a; Castella et al., 2018). Retrospective robust estimation of $R_{2}^{⋆}$ parameters (outlier rejection) has been suggested in Weiskopf et al. (2014) and could be implemented in future releases of the hMRI toolbox. Robustness must also be considered in parallel to the spatial resolution *versus* sufficient SNR level tradeoff to improve the sensitivity of the technique to small developmental changes and plasticity. Thus, spatially adaptive noise removal methods (Tabelow et al., 2016) along with appropriate handling of the Rician bias problem (Polzehl and Tabelow, 2016; Tabelow et al., 2017) are important future improvements of the hMRI-toolbox.

Currently, quality assessment (QA) is provided in the hMRI-toolbox via a set of indicative parameters that should be used at the user's discretion (Appendix D). Future work will focus on further validating and implementing an automated QA of the raw data and generated maps, providing figures from representative populations and protocols.

Extensions of the hMRI-toolbox can easily fit within its modular implementation. As short term future additions, the following three modules and extensions are considered. An additional module that efficiently calculates the protocol-specific correction parameters required to account for imperfect spoiling (Appendix A.5) is planned. Quantitative susceptibility mapping (QSM), taking advantage of the existing phase images acquired with the MPM protocol (Acosta-Cabronero and Callaghan, 2017; Acosta-Cabronero et al., 2018), is a second extension. Finally, as suggested by the ”h” in hMRI, adding new biophysical models that take advantage of the multi-contrast MRI data and generated qMRI maps for *in vivo* histology (Weiskopf et al., 2015) is another priority of future developments. An example for such a direct extension of the hMRI-toolbox could be the MR g-ratio model (Stikov et al., 2015; Mohammadi et al., 2015; Ellerbrock and Mohammadi, 2018). The MR g-ratio (the ratio between inner and outer diameter of a myelinated axon) is a geometrical microstructural tissue property that can be derived by combining myelin-sensitive qMRI maps from the hMRI-toolbox (e.g. MT or PD maps) with the axonal-sensitive maps obtained with existing SPM tools (e.g. the ACID toolbox (Mohammadi et al., 2012; Tabelow et al., 2015; Ruthotto et al., 2013; Mohammadi et al., 2014) and the DTI & Fiber tools (Reisert et al., 2013)).

## 5 Conclusion

The hMRI-toolbox is a highly flexible software package that provides a computationally time-efficient, robust and simple framework to generate and process qMRI parameter maps sensitive to myelin, iron, and water content. It profits from the powerful and easy-to-use spatial and statistical analysis tools in SPM, and can be readily combined with existing SPM tools for quantitative estimation of parameter maps sensitive to complementary information such as axonal properties. The ongoing developments address the use of open-science data formats and extensions into biophysical models for direct microstructure mapping. As such, the hMRI-toolbox is a comprehensive and readily extendable tool for estimating and processing qMRI data for neuroscience and clinical research.

## Figures and Tables

**Table 3:** JSON Metadata example: Metadata are stored as a Matlab structure in the JSON file associated to each output (NIfTI) image. The metadata structure can easily be retrieved using the following SPM/Matlab command: metadata = spm_jsonread(<name of the JSON file>). The structure includes processing parameters, dependencies of the result on other input images as well as a summary description of each output image. As an example, the image type of each output $R_{1}$ image used to generate Fig. 6 is provided in the Table. The highlighted lines (bold font) describe the $B_{1}$ transmit bias correction, illustrating the user-friendly traceability of the processing steps used to generate an image. The same information can be read directly from the JSON file using a text editor.

Output map	metadata.history.output.imtype
(A) $R_{1,Ref}$	R1 map [s-1]
	****- B1+ bias correction using provided B1 map (i3D_EPI)****
	- RF sensitivity bias correction based on a per-contrast sensitivity measurement
(B) $R_{1,UNICORT}$	R1 map [s-1]
	****- B1+ bias correction using UNICORT****
	- RF sensitivity bias correction based on a per-contrast sensitivity measurement
(C) $R_{1,None}$	R1 map [s-1]
	****- no B1+ bias correction applied****
	- RF sensitivity bias correction based on a per-contrast sensitivity measurement

**Table:** 

Source image	Recommended template
First T1w echo	SPM/canonical/avg152T1.nii
First PDw echo	SPM/canonical/avg152PD.nii
First MTw echo	SPM/canonical/avg152PD.nii

**Table 4:** Output files from the *Process hMRI maps* modules. The file names are based on the file name of the map or image used as input to the Segmentation, Dartel > Run Dartel (create Templates), Dartel > Normalise to MNI space, and Smoothing steps. Note that wc*<InputFileName>[nii|json]. images can be obtained either with the simple warp obtained with the *Unified Segmentation* or following Dartel with the Dartel > Normalise to MNI space module.

File name	Description
*Unified Segmentation (US)* (Proc. hMRI -> Segmentation)	
c1<segmInputFileName>.[nii|json]	Tissue class 1 in subject space. Prefixes c1/c2/c3 correspond to GM/WM/CSF respectively.
rc1<segmInputFileName>.[nii|json]	Tissue class 1 in subject space, resliced and imported for Dartel processing. Dartel usually relies on rc1/rc2 images (GM/WM) only.
wc1<segmInputFileName>.[nii|json]	Tissue class 1 warped into MNI space, with the simple warp obtained with the *US*. Prefixes wc1/wc2 correspond to GM/WM respectively.
mwc1<segmInputFileName>.[nii|json]	Tissue class 1 warped into MNI space, with the simple warp obtained with the *US*, and modulated by the determinant of the Jacobian, i.e. accounting for local change of volume. Prefixes mwc1/mwc2 correspond to GM/WM respectively.
y_<segmInputFileName>.nii	Deformation field, i.e. warps, obtained from *US*
<segmInputFileName>_seg8.mat	Segmentation and warping parameters, obtained from *US*
*Diffeomorphic registration* (Proc. hMRI -> Dartel -> Run Dartel (create Templates))	
u_rc1*_Template.nii	Flow field image, one per subject, estimated by Dartel from the rc1/rc2 images.
Template_*.nii	7 template images, numbered from 0 to 6, created by Dartel from the rc1/rc2 images of all the subjects.
*Diffeomorphic registration* (Proc. hMRI -> Dartel -> Normalise to MNI space)	
w*<normInputFileName>.[nii|json]	Image warped into MNI space following Dartel, using the estimated *flow field* and an affine transformation. This would be typically a qMRI map that should *not* be modulated to account for volume changes.
mw*<normInputFileName>.[nii|json]	Image warped into MNI space and modulated by the determinant of the Jacobian, i.e. accounting for local change of volume. This would typically be any tissue probability map (i.e. image with a measure whose total amount over the brain volume should be preserved) to be used after smoothing for a VBM analysis.
*Tissue-weighted smoothing* (Proc. hMRI -> Smoothing)	
wap1_<smooFileName>.[nii|json]	Tissue-weighted smoothing for tissue class 1.
s*<FileName>.[nii|json]	(Any) image smoothed with a standard Gaussian filter (here for comparison, *not* in hMRI).

## Appendix A Summary of the model for MPM

### Appendix A.1 $R_{1}$ and PD estimation

For a single excitation and echo time TE, the signal intensity *S* is given by the Ernst equation for an ideal spoiled gradient echo acquisition (Ernst and Anderson, 1966; Helms et al., 2008a):

(A.1) $$S=A\cdot \text{sin}\alpha \cdot \frac{1-e^{-R_{1}\cdot TR}}{1-\text{cos}\alpha \cdot e^{-R_{1}\cdot TR}}\cdot e^{-R_{2}^{⋆}\cdot TE}.$$

We denote the repetition time by TR and the flip angle by *α*. *A* is proportional to the equilibrium magnetisation and thus to the proton density PD (Helms et al., 2008a).

For fast estimation of the $R_{1}$ and *A* parameters from (A.1) and a single echo of a T1w and a PDw acquisition, or the mean across multiple echoes for increased SNR, Helms et al. (2008a) introduced a linearisation of its exponential terms for short TR (i.e. $R_{1}\cdot TR\ll 1$):

(A.2) $$e^{-R_{1}\cdot TR}\underline{∼}1-R_{1}\cdot TR$$

leading to, after averaging over echos,

(A.3) $$S\underline{∼}A^{⋆}\cdot \text{sin}\alpha \cdot \frac{R_{1}\cdot TR}{1-\text{cos}\alpha \cdot \left(1-R_{1}\cdot TR\right)}$$

with

(A.4) $$A^{⋆}=\frac{A}{\#echoes}\cdot \underset{TE}{\sum }e^{-R_{2}^{⋆}\cdot TE}$$

When *S* is the averaged signal over several echoes, the sum in Eq. (A.4) is calculated across all echoes included in the averaged signal. This introduces an $R_{2}^{⋆}$ bias which increases with the number of echoes and depends on the TE values included in the average. The $R_{2}^{⋆}$ bias in $A^{⋆}$ can be evaluated using the $R_{2}^{⋆}$ estimate and corrected for. Alternatively, *S* can be the extrapolated signal at TE=0, which is free from $R_{2}^{⋆}$ weighting. Both the echo averaging with $R_{2}^{⋆}$ correction method and the TE=0 extrapolation method are implemented and available in the toolbox, and perform similarly (Balteau et al., 2018). The toolbox extrapolates to a TE of 0 ms by default.

Using the approximation $\text{sin}\left(\alpha \right)\underline{∼}\alpha$ and $\text{cos}\left(\alpha \right)\underline{∼}1-\alpha^{2}/2$ for small flip angles and neglecting the term $\alpha^{2}\cdot R_{1}\cdot TR$, Eq. (A.1) simplifies to:

(A.5) $$S=A^{⋆}\cdot \alpha \cdot \frac{R_{1}\cdot TR}{\alpha^{2}/2+R_{1}\cdot TR}$$

from which $R_{1}$ and $A^{⋆}$ are estimated using the signals from the PDw and T1w sequences:

(A.6) $$R_{1}\underline{∼}\frac{S_{PD}\alpha_{PD}/TR_{PD}-S_{T1}\alpha_{T1}/TR_{T1}}{2\cdot \left(S_{T1}/\alpha_{T1}-S_{PD}/\alpha_{PD}\right)}$$

(A.7) $$A^{⋆}\underline{∼}\frac{S_{T1}S_{PD}\left(TR_{T1}\alpha_{PD}/\alpha_{T1}-TR_{PD}\alpha_{T1}/\alpha_{PD}\right)}{S_{PD}TR_{T1}\alpha_{PD}-S_{T1}TR_{PD}\alpha_{T1}}$$

where signals $S_{T1}$ and $S_{PD}$ are either averaged over a number of echoes (Helms et al., 2008a), or extrapolated to TE=0, as explained above. In the first case, a $\underset{TE}{\sum }e^{-R_{2}^{⋆}\cdot TE}$ correction factor must be calculated to derive *A* (Balteau et al., 2018). $A=A^{⋆}$ otherwise meaning that the $R_{2}^{⋆}$ bias remains.

### Appendix A.2 Estimation of the magnetisation transfer

The MT (magnetisation transfer) saturation measure is derived in analogy to a dual-excitation spoiled gradient echo sequence where the second excitation is replaced by the MT pulse. Using a heuristic approximation for small read-out flip angles $\alpha_{MT}$, the closed form solution for the acquired signal *S* can be written (Helms and Hagberg, 2009; Helms et al., 2008b) as

(A.8) $$S_{MT}=A\cdot \text{sin}\alpha_{MT}\cdot \frac{1-e^{-R_{1}\cdot TR_{MT}}-\delta \cdot \left(e^{-R_{1}\cdot TR_{2}}-e^{-R_{1}\cdot TR_{MT}}\right)}{1-\text{cos}\alpha_{MT}\cdot \left(1-\delta \right)\cdot e^{-R_{1}\cdot TR_{MT}}}\cdot e^{-R_{2}^{⋆}\cdot TE}$$

where $TR_{MT}=TR_{1}+TR_{2}$ is the total repetition time, and *δ* describes the MT saturation (more generally denoted MT in this paper).

From an additional MTw signal $S_{MT}$ we obtain, using again the linearisation of the exponential term for short $TR_{MT}$ (i.e. $R_{1}\cdot TR_{MT}\ll 1$) and the small angle approximation for the trigonometric functions:

(A.9) $$\delta =MT=\left(A^{⋆}\alpha_{MT}/S_{MT}-1\right)\cdot R_{1}\cdot TR_{MT}-\alpha_{MT}^{2}/2$$

see Helms et al. (2008b). Note that the parameter maps $R_{1}$, $R_{2}^{⋆}$, *A* and MT can alternatively be estimated from the ESTATICS model using analytic formulas and not relying on the linearisation outlined above (Tabelow et al., 2016).

### Appendix A.3 The ESTATICS model and $R_{2}^{⋆}$ estimation

The ESTATICS model was introduced in Weiskopf et al. (2014) for efficiently estimating the effective transverse relaxation rate $R_{2}^{⋆}$ from an MPM acquisition protocol, where the exponential signal decay with the echo time is identical for T1w, PDw and MTw acquisitions. Specifically, the signal equations (A.1), (A.2), (A.3), (A.4), (A.5), (A.6), (A.7), (A.8) are re-written as

(A.10) $$S=\left(S_{PD}\cdot I_{PD}+S_{T1}\cdot I_{T1}+S_{MT}\cdot I_{MT}\right)\cdot e^{-R_{2}^{⋆}\cdot TE}$$

with indicator variables $I_{PD}$, $I_{T1}$, and $I_{MT}$ for the differently weighted acquisitions. The signal intensities, e.g. $S_{PD}$, at TE=0 are given by

(A.11) $$S_{PD}=A\cdot \text{sin}\alpha_{PD}\cdot \frac{R_{1}\cdot TR_{PD}}{1-\text{cos}\alpha_{PD}\left(1-R_{1}\cdot TR_{PD}\right)}$$

with the flip angle and repetition time of the PDw sequence and correspondingly for $S_{T1}$ and $S_{MT}$. From Eq. (A.10) $R_{2}^{⋆}$ can be estimated using all echoes.

### Appendix A.4 Corrections for transmit and receive field inhomogeneities

Due to inhomogeneities of the $B_{1}$ transmit field the local flip angle deviates from its nominal value (Helms et al., 2008a). Using a correction field $f_{T}$, the effective local flip angle is given by

(A.12) $$\alpha_{corr}=f_{T}\cdot \alpha$$

For details on the correction field $f_{T}$ estimation see Section 3.7.2 and C.3.

For the MTw acquisition we denote by $\alpha_{sat}$ the off-resonance Gaussian shaped RF pulse used for MT weighting. The $B_{1}$ transmit bias field $f_{T}\left(x\right)$ leads to a local correction of $\alpha_{sat}$, too, and thus to a residual bias field for the estimated *δ* even though *δ* is largely independent of the local transmit field bias unlike other MT metrics such as MTR (Helms et al., 2008b; Callaghan et al., 2014). In Helms (2015) the heuristic correction factor for *δ* was found to be

(A.13) $$\frac{1-B\cdot \alpha_{sat}}{\left(1-B\cdot f_{T}\cdot \alpha_{sat}\right)\cdot f_{T}^{2}}$$

with $B\cdot \alpha_{sat}=0.4$ for the typically used value $\alpha_{sat}=220{}^{\circ}$ in MPM sequences (Weiskopf et al., 2013).

Finally, the multiplicative RF sensitivity bias field $f_{R}$ (see Section 3.7.3 and Appendix C.4) for the correction of the signal amplitude maps *A*:

(A.14) $$A_{corr}=A\cdot f_{R}$$

has to be measured or estimated from the data (for details see Section 3.7.3). The $A^{⋆}$ maps can then be normalised to proton density PD maps by calibration. To this end, the mean PD value in white matter is assumed to be 69 percent units (p.u.) (Filo and Mezer, 2018).

### Appendix A.5 Spoiling corrections

Further, imperfect spoiling requires an additional correction when estimating $R_{1}$ (Preibisch and Deichmann, 2009), to account for deviation from Ernst equation (A.1). The correction is performed using the following expression

(A.15) $$R_{1}^{corr}=\frac{R_{1}}{P_{a}\left(f_{T}\right)\cdot R_{1}+P_{b}\left(f_{T}\right)}$$

where $P_{a}\left(f_{T}\right)$ and $P_{b}\left(f_{T}\right)$ are quadratic functions in $f_{T}$. Their coefficients depend on the specific sequence and can be determined by simulation (Preibisch and Deichmann, 2009). Further refinements of this correction include diffusion effects on signal spoiling (Yarnykh, 2007; Lutti and Weiskopf, 2013; Callaghan et al., 2015c).

## Appendix B DICOM import and JSON metadata

To support the flexibility of the hMRI processing pipeline and the traceability of the data at every step, a number of parameters from acquisition to statistical results must be retrieved and stored.

Therefore, DICOM import and JSON metadata handling functionalities have been implemented with the following objectives:

1. Retrieving the full DICOM header as a readable and searchable Matlab structure (metadata),
2. During DICOM to NIfTI conversion, storing the metadata structure as separate JSON file (alongside the NIfTI image file) and/or as extended header into the NIfTI image file,
3. Handling metadata in order to set, get and search parameters.

With the current implementation, these objectives are achieved: the hMRI-toolbox handles and stores data acquisition and processing parameters as JSON-encoded metadata alongside brain imaging data sets. The implementation rests on the SPM12 implementation of DICOM tools (spm_dicom_header(s), spm_dicom_essentials, spm_dicom_convert) and relies on spm_jsonread and spm_jsonwrite for handling JSON-encoded metadata.

From SPM12 version r7219 (released 16 November 2017) and in later versions, functionalities described in points 1. and 2. above (excluding the extended header option though) have been integrated to the DICOM import module in SPM12 (implementation from the hMRI-toolbox). Note that some features of the hMRI-toolbox metadata library (for metadata handling, in particular to set, get and search the metadata) are not available in the SPM12 distribution.

Detailed syntax and many examples are provided in the hMRI-toolbox Wiki, we briefly describe the main features and the metadata structure below.

### Appendix B.1 DICOM to NIfTI conversion

The spm_dicom_convert script has been modified to achieve the above goals. An additional input argument has been set to deal with the new JSON metadata options. If JSON metadata storage is enabled, DICOM header information is stored as a JSON-formatted structure either as a separate JSON file or as extended NIfTI header (or both). If omitted or disabled, the DICOM to NIfTI conversion proceeds using exactly the same implementation as in the previously mentioned SPM12 distribution. The output NIfTI images, extended or not, have a valid NIfTI-1 format compatible with standard NIfTI readers (MRICron, SPM, FreeSurfer). Note that FSL up to version v5.0.9 does not support extended headers. For that reason, the extended header option, although suggested in (Gorgolewski et al., 2016) is currently not recommended and disabled by default. Instead, a separate JSON file is created.

### Appendix B.2 DICOM to NIfTI conversion – SPM Batch.

The DICOM to NIfTI conversion with the above features is available through the hMRI-toolbox in the SPM Batch GUI (SPM > Tools > hMRI Tools > DICOM Import). The new JSON metadata format field provides the user with the following options:

• None
• Separate JSON file (default)
• Extended nii header
• Both

### Appendix B.3 The metadata structure.

Metadata are simple MATLAB structures, hence quite flexible and modular. The structure of the metadata is divided into the following two main fields:

• Acquisition parameters (**hdr.acqpar**): this branch contains the *original DICOM header.* It should be kept unchanged and is created when importing DICOM images to NIfTI. It is dropped for processed images relying on several input images.
• History (**hdr.history**): is a *nested* structure containing
  – **procstep**: a structure describing the current processing step and containing:
    ∗ **descrip**: a brief description of the processing applied (e.g. DICOM to NIfTI conversion, realign, create map)
    ∗ **version**: version number of the processing applied, for traceability
    ∗ **procpar**: processing parameters (relevant parameters used to process the data – e.g. for SPM processing, all the settings included in the MATLAB batch job except for the input images.
  – **input.** An array listing the input images used for the processing. Each input (hdr.history.input(i)) is a structure containing:
    ∗ **filename**: the filename of the input image
    ∗ **history**: the history structure from the input image
  – **output**: a structure containing
    ∗ **imtype**: image type of the current output (e.g. $R_{1}$, FA, MT, ADC) including a summary description of the processing steps (see TableB 3).
    ∗ **units**: either physical units (e.g. sec, 1/sec), standardised units (e.g. percent units = p.u.) or arbitrary units (a.u.).

### Appendix B.4 Metadata handling

The JSON transcription of the MATLAB structure is easily readable and searchable by opening the extended NIfTI or separate JSON file with a text editor. Furthermore, metadata set, get and search tools have been implemented. These tools have been added into the spm12/metadata directory. Typing help <function> in MATLAB gives detailed syntax. Below is a list of the most useful scripts and their summary descriptions. For a complete description of the implemented tools, please refer to the hMRI-toolbox Wiki pages.

get_metadata: returns the MATLAB structure described above.

set_metadata: to write (insert, modify, overwrite) metadata in JSON files and extended NIfTI headers. In general, metadata are initialised during the DICOM to NIfTI conversion and further modified as the processing progresses.

get_metadata_val: returns pairs of structure fields and values agreeing with search criteria.

find_field_name: recursively searches a MATLAB structure for a specific field name (can be applied to any MATLAB structure).

### Appendix B.5 General usage of the metadata and BIDS compatibility

The DICOM import with metadata storage is available in the SPM distribution since release r7219 (November 2017) allowing for a wide use of such metadata. It provides most of the parameters required for map creation and processing. The hMRI-toolbox also provides metadata handling functionalities to retrieve parameter values and store processing parameters in the JSON-encoded metadata file. The format used for the metadata provides flexibility for using a wide range of protocols, and traceability of every parameter used to generate the results (see for example Table 3).

Outside the hMRI-toolbox, metadata and metadata handling tools implemented in the hMRI-toolbox can prove useful for many purposes. For quality control, metadata can help checking for protocol consistency by comparing essential parameters across sessions, subjects and sites. When processing data acquired with several protocols on several scanners from different vendors (multi-centre studies), metadata provide the information to automatically extract acquisition parameters to be used as regressors of no interest in a statistical analysis (e.g. MR scanner, field strength, head coil, …). Metadata also facilitate the sorting of the data between different data types (e.g. functional MRI, structural MRI, diffusion images, etc.) together with retrieving BIDS essential parameters (e.g. repetition time, echo time, flip angle, session and series numbers, etc.), therefore providing all the information required to make the data archiving and storage fully BIDS-compatible.

A *BIDS extension proposal (BEP)* is currently under discussion, that will define the parameters necessary to describe *structural acquisitions with multiple contrasts* (see https://bids.neuroimaging.io/for updates on BIDS specifications and extension proposals), and bring the metadata structure of such data in line with the BIDS recommendations (Gorgolewski et al., 2016). The extension will include all acquisition parameters required to fully describe and process data acquired with the MPM protocols, in a standard, platform- and vendor-independent way. With such BIDS extension, the hMRI-toolbox will be adapted and made fully BIDS-compliant, from the structure of the JSON-encoded metadata to the structure of the output data. The required adaptation will be achieved smoothly thanks to the metadata handling functionalities, without affecting the processing part of the hMRI-toolbox, and enabling the implementation of fully automated tools retrieving the BIDS-organised data, generating the maps and applying spatial processing.

## Appendix C Detailed description of the hMRI modules

### Appendix C.1 Configure toolbox module

The *Configure toolbox* module must be run before any other module for the default parameters to take effect.

The default settings are defined and described in the config directory. The default files therein should never be modified. Template files for customised default parameters are stored in the config/local directory and can be modified and saved with meaningful names by the user. Note that most parameters should only be modified by advanced users who are aware of the underlying theory and implementation of the toolbox.

For brevity, a full description of each customisable setting is not provided here. A detailed description of each parameter may be found in the hmri_local_defaults.m help and examples are provided in the toolbox Wiki pages. Note that the default settings for $B_{1}$ transmit bias correction (also including acquisition and processing parameters) can be customised separately within the *Create hMRI maps* module (Appendix C.3). Their customisation must follow the same guidelines as provided in this section.

While settings need to be defined either in the standard or customised default files, acquisition parameters can be retrieved from the input images if included in the metadata. When no metadata are available or not yet properly handled by the toolbox (e.g. customised sequences), acquisition parameters specified in the default files are used as a fallback solution. The use of metadata is strongly recommended (see Appendix B), to automatically retrieve acquisition parameters and to avoid incorrect processing.

### Appendix C.2 Auto-reorient module

This module performs a rigid-body alignment of a subject's structural image into the MNI space, applying the estimated pose to a set of *other images*. The code makes use of the spm_affreg function and *canonical* images available in SPM12. The user must provide one structural image from the subject and a corresponding canonical image for co-registration, and a series of other images that need to be kept in alignment with the structural image (typically, all the images acquired during a given imaging session). The structural image should have good signal-to-noise ratio with a high white matter/grey matter contrast-to-noise to ensure a robust and reliable co-registration. The SPM canonical images can be selected from the SPM/canonical directory or any other user-defined template (e.g. atypical population-driven template), provided it is already oriented according to MNI. Since the registration is based on matching similar intensities, it is important that the contrast of the reference image be close to that of the canonical image. The following list is provided as guidelines:

If no specific output directory is selected, the original orientation of the images will be overwritten. If an output directory is selected then the images are copied there before being reoriented, therefore leaving the original data untouched. The reoriented images can be collected as dependencies for further processing in the batch interface, either all at once (Dependencies > Grouped) or as individual output images (Dependencies > Individual), which is more convenient to connect the *Auto-reorient* module to the next *Create hMRI maps* module.

### Appendix C.3 B_1_ transmit field corrections

Within the *Create hMRI maps* module, several methods for $B_{1}$ transmit field correction are implemented. Depending on the choice of the specific method the GUI requires the user to provide adequate input files, which are described below. In order to proceed to the $B_{1}$ map calculation, number of acquisition parameters must also be either retrieved from the metadata or provided to the toolbox via a standard or customised default $B_{1}$ file (see below and in Appendix C.1).

1. 3D_EPI

EPI spin-echo (SE)/stimulated-echo (STE) method (Lutti et al., 2009, 2012).

The sequence uses a set of pulses with nominal flip angles *α*, 2⋅α and *α* to create pairs of spin echo and stimulated echo images. All consecutive pairs of SE/STE images corresponding to the different nominal flip angle values *α* of the SE/STE RF pulse must be loaded as B1 input. $B_{0}$ field mapping images must also be provided for correcting distortions in the EPI images. Both magnitude images and the pre-subtracted phase image must be selected, in that order, as B0 input.

2. 3D_AFI

*Actual Flip Angle Imaging (AFI)* method (Yarnykh, 2007).

A pair of magnitude images acquired with two different repetition times (TR) must be loaded as B1 input. The hMRI-toolbox then calculates the $B_{1}$ transmit bias field map as described in Yarnykh (2007).

3. tfl_b1_map

TFL $B_{1}$ mapping (Chung et al., 2010).

For this method, the batch interface requests a pair of images (one anatomical image and one flip angle map, in that order) from a service sequence by Siemens (version available from VE11 on) based on a turbo flash (TFL) sequence with and without a pre-saturation pulse (Chung et al., 2010). The flip angle map used as input contains the measured flip angle multiplied by 10. After rescaling (p.u.) and smoothing, the output $f_{T}$ map is ready to be used for $B_{1}$ transmit bias correction.

4. Rf_map

This requires a pair of images (one anatomical image and one pre-processed $B_{1}$ map, in that order) from a service sequence by Siemens (Erlangen, Germany). These are based on the acquisition of a spin-echo/stimulated echo as used for method 3D_EPI above (Lutti et al., 2009, 2012). Rescaling and smoothing is applied to the input $B_{1}$ map (a.u.) to generate a $f_{T}$ map (p.u.) suitable for $B_{1}$ transmit bias correction.

5. Pre-processed B1

Any $B_{1}$ transmit bias field map pre-calculated using one of the above methods or another method can be used as pre-processed B1 input. The user must select one anatomical reference (for co-registration) and one $B_{1}$ map (in p.u.), in that order. The anatomical reference is typically a magnitude image from the $B_{1}$ mapping dataset, and must be in the same space as the $B_{1}$ transmit bias field map.

6. UNICORT

Data driven estimation of the $B_{1}$ transmit bias field map using the UNICORT approach (Weiskopf et al., 2011).

If none of the above mentioned methods applies because no appropriate $B_{1}$ transmit bias field mapping data were acquired, the UNICORT option is recommended to remove the transmit field bias in the $R_{1}$ map and estimate the $B_{1}$ transmit bias field map from the qMRI data. Optimal UNICORT processing parameters are RF transmit coil dependant. The default parameters have been optimised for Siemens 3T TIM Trio scanners and may need specific optimisation for other RF transmit coils. See the hMRI-toolbox Wiki for guidelines.

7. No_B1_correction

With this option, no correction for transmit inhomogeneities is applied, i.e. $f_{T}\equiv 1$.

For each $B_{1}$ mapping protocol (1–4), one *_B1map (in p.u.) and one *_B1ref (for anatomical reference) file is created and saved in the Results/Supplementary directory (see Table 2). The output images are associated with JSON metadata including the type of $B_{1}$ map processed and the setting used. For UNICORT, only the *_B1map in p.u. Is created (in the same space as the qMRI maps) with no anatomical reference. For pre-processed $B_{1}$ transmit bias field maps provided by the user, the input files are not renamed to match the naming convention above. Default acquisition and processing parameters for the UNICORT, 3D_EPI and 3D_AFI methods can be customised at this point by providing the *Create hMRI maps* module with a customised configuration file (see Appendix C.1 and the hMRI Wiki pages for guidelines). Except for UNICORT, where the $B_{1}$ transmit bias field is directly derived from the qMRI data, and the no_B1_correction option, where no anatomical reference is given, the hMRI-toolbox co-registers the anatomical reference image with the multi-echo spoiled gradient echo data before applying the $B_{1}$ transmit bias correction.

### Appendix C.4 Receive (RF) sensitivity bias correction

In this paragraph, the RF sensitivity bias correction methods based on measured RF sensitivity maps (Single or Per contrast options) as well as the data driven method (Unified Segmentation option: no input sensitivity map required) will be explained in detail.

*Receive field sensitivity measurements*. Since the receive field sensitivity of the body coil is assumed to be flat over the spatial extent of the head (Pruessmann et al., 1999), if the same anatomy is imaged with the head coil and the body coil sequentially, using the same acquisition parameters and assuming no motion, then the ratio of these two scans is proportional to the net head coil receive sensitivity field $f_{R}$:

(C.1) $$S_{HC}=f_{R}S_{0};S_{BC}=C_{BC}S_{0}$$

(C.2) $$S_{HC}/S_{BC}=f_{R}/C_{BC}$$

where $S_{HC}$ is the signal acquired with the head coil, $f_{R}$ is the receive sensitivity field of the head coil, $S_{BC}$ is the same signal acquired by the body coil, $C_{BC}$ is the receive sensitivity field of the body coil assumed to be approximately constant, and $S_{0}$ is the signal specific to the underlying anatomy and acquisition parameters.

If receive field sensitivity measurements are available for each imaging contrast (one receive sensitivity field acquired either before or after each of the PDw, T1w and MTw contrasts), inter-scan variation of the sensitivity modulation can be accounted for and used to optimally correct for the combined inter-scan motion and sensitivity modulation effects in the quantitative maps (Papp et al. (2016) and Fig. 5a). Therefore, the implemented RF sensitivity correction method (Per contrast option) combines a correction for motion-related relative receive sensitivity variations with rigid-body realignment.

The low resolution measurements from the head and body coils (in this order) for each T1w, PDw and MTw acquisition are expected as inputs to the RF sensitivity bias correction. A receive field sensitivity map is calculated according to Eqs. (A.1), (C.1), (C.2).2 for each contrast, and applied to the corresponding contrast to correct for any coil sensitivity driven signal intensity modulation.

If RF sensitivity measurements are missing for some of the contrasts, or if only one single measurement is available, the same RF sensitivity bias correction can still be applied to all contrasts by choosing the corresponding (Single) option in the GUI. Only one pair of head coil and body coil images is required. Receiver field inhomogeneities will be corrected for although not accounting for inter-scan motion (Fig. 5b–d). Note that the measured RF sensitivity maps are modulated by the receive field of the body coil, which is not corrected for (Fig. 5e).

*Data driven receive field estimation*. If no receive field sensitivity map has been acquired, the Unified Segmentation option can be selected in the GUI. The receive field bias is then estimated from the calculated *A* map using SPM unified segmentation (US) approach (Ashburner and Friston, 2005). Accurate bias field estimation requires the use of a brain mask combining the white and grey matter probability maps. The latter are derived by segmentation of the calculated MT map (because of its higher contrast in the basal ganglia, see Helms et al. (2009)) or alternatively (if no MTw images are available), by segmentation of the calculated $R_{1}$ map. The optimal US parameters depend on the RF receive coil and its sensitivity profile. Fine adjustment of the US parameters might be required for a specific RF receive coil.

### Appendix C.5 Process hMRI maps module

This section describes the three main operational steps of the spatial processing pipeline for hMRI data: segmentation, diffeomorphic registration and tissue-weighted smoothing. Furthermore, it provides details on the fully integrated processing pipeline, which is proposed in this toolbox as a separate sub-module to facilitate standard spatial data processing without the need to combine the individual steps.

The processing tools and user interface can be used to process parameter maps generated from a series of subjects, assuming that each subject has the same small number of images (for example the four MT saturation, PD, $R_{1}$, and $R_{2}^{⋆}$ maps). Therefore each module takes as input several series of images sorted per image type (for example the MT from all the subjects). The images in each series must follow the same subject-based ordering.

#### Appendix C.5.1 Segmentation

This step relies on the latest unified segmentation (US) algorithm as available in SPM12 (Ashburner and Friston, 2005) and is interfaced through a single module. Compared to the *SPM12-Segment* module, the *hMRI-Segment* module allows the successive processing of a series of subjects.

The segmentation itself relies on one structural image per subject, typically an MT map, $R_{1}$ map or T1w image, and a set of tissue probability maps (TPMs). The TPMs used by default in the hMRI-toolbox were specifically derived from multi-parametric maps (Lorio et al., 2014) denoted as *extended TPMs* (eTPM).

By default (adjustable in the batch interface), the segmentation step generates a series of tissue class images in native space: the c1/c2/c3 images - for gray matter (GM), white matter (WM) and CSF, as well as Dartel-imported rc1/rc2 images. The segmentation module also outputs the GM and WM tissue class images warped into MNI space (classic elastic deformation, i.e. *not* with Dartel) with (wmc1/wmc2 images) and without (wc1/wc2 images) Jacobian modulation. Finally, the estimated segmentation and deformation fields are available as a.mat file and 4D y_*.nii files respectively. See the upper part of Table 4 for a complete file list and description of the output.

#### Appendix C.5.2 Diffeomorphic registration

This step includes three sub-modules derived from the Dartel toolbox (Ashburner, 2007). The key idea of Dartel is to iteratively align the tissue class images, typically the grey and white matter, from a series of subjects to their own average, i.e. one per tissue class considered, by generating successive mean images with higher overlap and detail (called Templates). This only depends on the Dartel-imported tissue class images from each subject.

The three sub-modules included are as follows:

*Run Dartel, create Templates*. This module aligns together tissue class images from the series of subjects. In addition a set of Templates (population average tissue class images, with increasing sharpness), this sub-module creates a deformation field for each subject, which encodes the nonlinear alignment with the final template. See the second part of Table 4.

*Run Dartel, existing Templates*. If the set of Templates already exists, this sub-module can be used to estimate only the deformation fields for another set of new subjects (relying on the same form of tissue class images as those used to generate the Templates).

*Normalize to MNI*. This module applies the estimated deformation fields to the parameter maps and tissue class images, bringing them all into alignment with MNI space. Unlike to the Dartel toolbox, no smoothing is applied at this stage, but a specific smoothing operation is applied at the next *Tissue-weighted smoothing* step. See the third part of Table 4 for the file list and description.

The machinery behind those modules are exactly the same as the ’Dartel toolbox’ available elsewhere in SPM. They have only been included in the hMRI-toolbox for ease of use. Moreover the user-interface of the *Normalize to MNI* module was slightly extended to more efficiently handle the multiple parameter maps and tissue class images of each subject. Notably, in contrast to many typical VBM applications, quantitative parameter values are not modulated (to account for volume changes) during this normalization.

#### Appendix C.5.3 Tissue-weighted smoothing

As proposed in Draganski et al. (2011), the parameter maps should be smoothed to improve spatial realignment while accounting for the partial volume contribution of the tissue density in each voxel in subject space. This combination of weighting and smoothing is expressed as:

(C.3) $$v=\frac{g*\left(w\;s\left(\phi \right)\left[eTPM\left(t\right)>.05\right]\right)}{g*w}\left[g*w\;>\;.05\right]$$

where:

• *v* is the resulting *tissue-weighted smoothed* image;
• g∗ stands for convolution with a Gaussian smoothing kernel;
• *ϕ* encodes the spatial deformation from subject to standard space;
• s(ϕ) is a parameter map (e.g. MT, $R_{1}$, …) in standard space warped by *ϕ*;
• w=|Dϕ|t(ϕ), the weights representing the tissue class density in standard space, accounting for volume changes;
• t(ϕ) represents subject's tissue class image, typically GM or WM, warped by *ϕ*;
• |Dϕ| is a Jacobian determinants of deformation *ϕ*;
• [x>0.05] is a masking operation where voxels with a value *x* smaller than .05 are set to zero.

The masking ensures that only voxels with a) an *a priori* probability of being in the considered tissue class above 5% (i.e. the whole intra-cranial brain volume for GM and WM) and b) original tissue density in native space above 5% are included in the resulting smoothed image.

One *tissue-weighted smoothed* map (*v*) is generated per tissue class (*t*) and parameter map (*s*). These images can then be statistically analysed. See the bottom part of Table 4 for the file prefix list and description. Note, that the tissue-weighted smoothed maps for different tissue classes will spatially overlap, because they were thresholded at 5%. Thus, additional masking of these tissue-specific maps at the group-level may be desirable to ensure that a given voxel is analysed only once. Also note, that the VBQ smoothing, in contrast to typical VBM analyses, does not modulate the parameter maps (to account for volume changes), but instead preserves the quantitative values of the original qMRI maps in MNI space.

#### Appendix C.5.4 Integrated pipeline

Spatial processing of multiple parameter maps would typically combine four modules (*Segmentation*, *Run Dartel, create Templates*, *Normalize to MNI*, and finally *Tissue-weighted smoothing*) into a single batch. The options of each module can be adjusted and the images passed from one module to the next via virtual dependencies, as available in the batch-GUI, forming a complete processing pipeline. An alternative approach, simpler but less spatially accurate (not recommended), would skip the diffeomorphic warping steps and only combine the *Segmentation* and *Tissue-weighted smoothing* modules.

To help novice users, an *Integrated pipeline* module directly enacts the whole procedure with minimal user input. The module only requires the following inputs:

• the series of structural reference images and parameter maps (one per subject),
• the width of the Tissue-weighted smoothing kernel desired,
• the type of processing pipeline, i.e. with or without diffeomorphic registration (Dartel).

All the other options of each of the intermediate steps are set by defaults, e.g. only GM and WM tissue classes are of interest and the TPMs are those from the hMRI-toolbox.

## Appendix D Quantitative maps and quality assessment

The Quality Assessment (QA) tool provided with the *Create hMRI maps* module generates relative indicators of the quality of the quantitative maps. This tool is enabled via the QA flag in the hmri_defaults.m file and can be modified using a local configuration (Appendix C.1). When enabled, a JSON-encoded MATLAB structure containing objective measures of the quality of the data is saved in the Results/Supplementary directory (Table 2). The structure contains the following fields:

1. Coregistration parameters of the MTw and T1w images to the PDw images (translation in mm and rotation in radians), providing measures of head motion between the corresponding images (*inter-scan motion*).
2. Standard deviation in white matter of the $R_{2}^{⋆}$ maps (SD-$R_{2}^{⋆}$), calculated from each individual multi-echo acquisition (PDw, T1w, MTw) and providing measures of image degradation due to head motion during the acquisition of the corresponding echoes (*intra-scan motion*) (Castella et al., 2018). NOTE: SD-$R_{2}^{⋆}$ depends on the noise level in the images (related to image resolution) and on the number of echoes for each (PDw, T1w, MTw) contrast, affecting the robustness of the $R_{2}^{⋆}$ fit. Moreover, since $R_{2}^{⋆}$ is a marker of the microstructural properties of brain tissue, microscopic changes in the brain may affect the SD-$R_{2}^{⋆}$ values.
3. Mean and standard deviation of the PD values in white matter, which ratio (standard deviation/mean) reflects the accuracy of the $B_{1}^{-}$ (RF receive sensitivity) bias field correction in healthy subjects (Papp et al., 2016). NOTE: pathological inhomogeneities of brain tissue (e.g. brain lesions) may contribute and alter the predictability of this parameter.

These parameters are population- and protocol-dependent which is why they are referred to as relative indicators.

## References

[bib1] Acosta-Cabronero J., Callaghan M.F. (2017). Quantitative susceptibility mapping from variable flip angle measurements. ESMRMB 2017.

[bib2] Acosta-Cabronero J., Milovic C., Mattern H., Tejos C., Speck O., Callaghan M.F. (2018). A robust multi-scale approach to quantitative susceptibility mapping. NeuroImage.

[bib3] Alonso-Ortiz E., Levesque I.R., Pike G.B. (2018). Impact of magnetic susceptibility anisotropy at 3T and 7T on T2*-based myelin water fraction imaging. Neuroimage.

[bib4] Ashburner J. (2007). A fast diffeomorphic image registration algorithm. Neuroimage.

[bib5] Ashburner J., Friston K.J. (2000). Voxel-based morphometry–the methods. Neuroimage.

[bib6] Ashburner J., Friston K.J. (2005). Unified segmentation. Neuroimage.

[bib7] Assaf Y., Basser P.J. (2005). Composite hindered and restricted model of diffusion (CHARMED) MR imaging of the human brain. Neuroimage.

[bib8] Balteau E., Leutritz T., Weiskopf N., Reimer E., Lutti A., Callaghan M.F., Mohammadi S., Tabelow K. (2018). Evaluating T2* bias impact and correction strategies in quantitative proton density mapping. Proceedings of ISMRM 2018.

[bib9] Battiston M., Cercignani M. (2018). MT: magentisation transfer.

[bib10] Baudrexel S., Reitz S.C., Hof S., Gracien R.-M., Fleischer V., Zimmermann H., Droby A., Klein J.C., Deichmann R. (2016). Quantitative T1 and proton density mapping with direct calculation of radiofrequency coil transmit and receive profiles from two-point variable flip angle data. NMR Biomed..

[bib11] Bernstein M.A., King K.F., Zhou X.J. (2004). Handbook of MRI Pulse Sequences.

[bib12] Büchel C., Raedler T., Sommer M., Sach M., Weiller C., Koch M.A. (2004). White matter asymmetry in the human brain: a diffusion tensor MRI study. Cerebr. Cortex.

[bib13] Cabana J.-F., Gu Y., Boudreau M., Levesque I.R., Atchia Y., Sled J.G., Narayanan S., Arnold D.L., Pike G.B., Cohen-Adad J. (2015). Quantitative magnetization transfer imaging made easy with qMTLab: software for data simulation, analysis, and visualization. Concepts Magn. Reson. Part A Bridg. Educ. Res..

[bib14] Callaghan M.F., Dick F., Grabher P., Keller T., Freund P., Weiskopf N. (2016). Optimisation of post-processing correction of transmit field inhomogeneity in R1 maps by relaxometry modelling. Proceedings of ISMRM 2016.

[bib15] Callaghan M.F., Freund P., Draganski B., Anderson E., Cappelletti M., Chowdhury R., Diedrichsen J., Fitzgerald T.H.B., Smittenaar P., Helms G., Lutti A., Weiskopf N. (2014). Widespread age-related differences in the human brain microstructure revealed by quantitative magnetic resonance imaging. Neurobiol. Aging.

[bib16] Callaghan M.F., Helms G., Lutti A., Mohammadi S., Weiskopf N. (2015). A general linear relaxometry model of R1 using imaging data. Magn. Reson. Med..

[bib17] Callaghan M.F., Josephs O., Herbst M., Zaitsev M., Todd N., Weiskopf N. (2015). An evaluation of prospective motion correction (PMC) for high resolution quantitative MRI. Front. Neurosci..

[bib18] Callaghan, M.F., Lutti, A., Ashburner, J., Balteau, E., Corbin, N., Draganski, B., Helms, G., Kherif, F., Leutritz, T., Mohammadi, S., Phillips, C., Reimer, E., Ruthotto, L., Seif, M., Tabelow, K., Ziegler, G., Weiskopf, N., submitted. Example dataset for the hMRI toolbox. Data In Brief. 10.1016/j.dib.2019.104132 PMC6598837 31297422

[bib19] Callaghan M.F., Malik S.J., Weiskopf N. (2015). Rapid calculation of correction parameters to compensate for imperfect RF spoiling in quantitative R1 mapping. Proceedings of ISMRM 2015.

[bib20] Callaghan M.F., Mohammadi S., Weiskopf N. (2016). Synthetic quantitative MRI through relaxometry modelling. NMR Biomed..

[bib21] Canna A., Ponticorvo S., Russo A.G., Manara R., Di Salle F., Saponiero R., Callaghan M.F., Weiskopf N., Esposito F. (Dec 2018). A group-level comparison of volumetric and combined volumetric-surface normalization for whole brain analyses of myelin and iron maps. Magn. Reson. Imaging.

[bib23] Carey D., Krishnan S., Callaghan M.F., Sereno M.I., Dick F. (2017). Functional and quantitative MRI mapping of somatomotor representations of human supralaryngeal vocal tract. Cerebr. Cortex.

[bib22] Carey D., Caprini F., Allen M., Lutti A., Weiskopf N., Rees G., Callaghan M.F., Dick F. (2018). Quantitative MRI provides markers of intra-, inter-regional, and age-related differences in young adult cortical microstructure. Neuroimage.

[bib24] Castella R., Arn L., Dupuis E., Callaghan M.F., Draganski B., Lutti A. (2018). Controlling motion artefact levels in MR images by suspending data acquisition during periods of head motion. Magn. Reson. Med..

[bib25] Cercignani M., Dowell N.G., Tofts P.S. (2018). Quantitative MRI of the Brain: Principles of Physical Measurement.

[bib26] Chenevert T.L., Malyarenko D.I., Newitt D., Li X., Jayatilake M., Tudorica A., Fedorov A., Kikinis R., Liu T.T., Muzi M., Oborski M.J., Laymon C.M., Li X., Thomas Y., Jayashree K.-C., Mountz J.M., Kinahan P.E., Rubin D.L., Fennessy F., Huang W., Hylton N., Ross B.D. (2014). Errors in quantitative image analysis due to platform-dependent image scaling. Transl. Oncol..

[bib27] Chung S., Kim D., Breton E., Axel L. (2010). Rapid B1+ mapping using a preconditioning RF pulse with TurboFLASH readout. Magn. Reson. Med..

[bib28] Dale A.M., Sereno M.I. (1993). Improved localizadon of cortical activity by combining EEG and MEG with MRI cortical surface reconstruction: a linear approach. J. Cognit. Neurosci..

[bib29] Davatzikos C., Bryan N. (1996). Using a deformable surface model to obtain a shape representation of the cortex. IEEE Trans. Med. Imag..

[bib30] Deichmann R., Schwarzbauer C., Turner R. (2004). Optimisation of the 3D MDEFT sequence for anatomical brain imaging: technical implications at 1.5 and 3 T. Neuroimage.

[bib31] Deoni S.C. (2007). High-resolution T1 mapping of the brain at 3T with driven equilibrium single pulse observation of T1 with high-speed incorporation of RF field inhomogeneities (DESPOT1-HIFI). J. Magn. Reson. Imag..

[bib32] Deoni S.C., Williams S.C., Jezzard P., Suckling J., Murphy D.G., Jones D.K. (2008). Standardized structural magnetic resonance imaging in multicentre studies using quantitative T1 and T2 imaging at 1.5 T. Neuroimage.

[bib33] Deoni S.C.L., Peters T.M., Rutt B.K. (2005). High-resolution T1 and T2 mapping of the brain in a clinically acceptable time with DESPOT1 and DESPOT2. Magn. Reson. Med..

[bib34] Dick F., Tierney A.T., Lutti A., Josephs O., Sereno M.I., Weiskopf N. (2012). In vivo functional and myeloarchitectonic mapping of human primary auditory areas. J. Neurosci..

[bib35] Dick F.K., Lehet M.I., Callaghan M.F., Keller T.A., Sereno M.I., Holt L.L., Nov (2017). Extensive tonotopic mapping across auditory cortex is recapitulated by spectrally directed attention and systematically related to cortical myeloarchitecture. J. Neurosci..

[bib36] Does M.D. (2018). Inferring brain tissue composition and microstructure via MR relaxometry. NeuroImage.

[bib37] Donahue K.M., Burstein D., Manning W.J., Gray M.L. (1994). Studies of Gd-DTPA relaxivity and proton exchange rates in tissue. Magn. Reson. Med..

[bib38] Draganski B., Ashburner J., Hutton C., Kherif F., Frackowiak R., Helms G., Weiskopf N. (2011). Regional specificity of MRI contrast parameter changes in normal ageing revealed by voxel-based quantification (VBQ). Neuroimage.

[bib39] Drury H.A., Van Essen D.C., Anderson C.H., Lee C.W., Coogan T.A., Lewis J.W. (1996). Computerized mappings of the cerebral cortex: a multiresolution flattening method and a surface-based coordinate system. J. Cognit. Neurosci..

[bib40] Edwards L.J., Kirilina E., Mohammadi S., Weiskopf N. (2018). Microstructural imaging of human neocortex in vivo. NeuroImage.

[bib41] Ellerbrock I., Mohammadi S. (2018). Four in vivo g-ratio-weighted imaging methods: comparability and repeatability at the group level. Hum. Brain Mapp..

[bib42] Ernst R.R., Anderson W.A. (1966). Application of Fourier transform spectroscopy to magnetic resonance. Rev. Sci. Instrum..

[bib43] Fatouros P.P., Marmarou A. (1999). Use of magnetic resonance imaging for in vivo measurements of water content in human brain: method and normal values. J. Neurosurg..

[bib44] Fatouros P.P., Marmarou A., Kraft K.A., Inao S., Schwarz F.P. (1991). In vivo brain water determination by T1 measurements: effect of total water content, hydration fraction, and field strength. Magn. Reson. Med..

[bib45] Filo S., Mezer A.A. (2018). PD: proton density of tissue water.

[bib46] Fischl B., Dale A.M. (2000). Measuring the thickness of the human cerebral cortex from magnetic resonance images. Proc. Natl. Acad. Sci. U.S.A..

[bib47] Fischl B., Sereno M.I., Tootell R.B., Dale A.M. (1999). High-resolution intersubject averaging and a coordinate system for the cortical surface. Hum. Brain Mapp..

[bib48] Focke N.K., Helms G., Kaspar S., Diederich C., Tóth V., Dechent P., Mohr A., Paulus W. (2011). Multi-site voxel-based morphometry–not quite there yet. Neuroimage.

[bib49] Freund P., Weiskopf N., Ashburner J., Wolf K., Sutter R., Altmann D.R., Friston K., Thompson A., Curt A. (2013). MRI investigation of the sensorimotor cortex and the corticospinal tract after acute spinal cord injury: a prospective longitudinal study. Lancet Neurol..

[bib50] Fullerton G.D., Potter J.L., Dornbluth N.C. (1982). NMR relaxation of protons in tissues and other macromolecular water solutions. Magn. Reson. Imaging.

[bib51] Gelman N., Ewing J.R., Gorell J.M., Spickler E.M., Solomon E.G. (2001). Interregional variation of longitudinal relaxation rates in human brain at 3.0 T: relation to estimated iron and water contents. Magn. Reson. Med..

[bib52] Gorgolewski K.J., Auer T., Calhoun V.D., Craddock R.C., Das S., Duff E.P., Flandin G., Ghosh S.S., Glatard T., Halchenko Y.O., Handwerker D.A., Hanke M., Keator D., Li X., Michael Z., Maumet C., Nichols B.N., Nichols T.E., Pellman J., Poline J.-B., Rokem A., Schaefer G., Sochat V., Triplett W., Turner J.A., Varoquaux G., Poldrack R.A. (2016). The brain imaging data structure, a format for organizing and describing outputs of neuroimaging experiments. Sci. Data.

[bib53] Helbling S., Teki S., Callaghan M.F., Sedley W., Mohammadi S., Griffiths T.D., Weiskopf N., Barnes G.R. (2015). Structure predicts function: combining non-invasive electrophysiology with in-vivo histology. Neuroimage.

[bib54] Helms G. (2015). Correction for residual effects of B1+ inhomogeniety on MT saturation in FLASH-based multi-parameter mapping of the brain. Proceedings of ISMRM.

[bib55] Helms G., Dathe H., Dechent P. (2008). Quantitative FLASH MRI at 3T using a rational approximation of the Ernst equation. Magn. Reson. Med..

[bib56] Helms G., Dathe H., Dechent P. (2010). Modeling the influence of TR and excitation flip angle on the magnetization transfer ratio (MTR) in human brain obtained from 3D spoiled gradient echo MRI. Magn. Reson. Med..

[bib57] Helms G., Dathe H., Kallenberg K., Dechent P. (2008). High-resolution maps of magnetization transfer with inherent correction for RF inhomogeneity and T1 relaxation obtained from 3D FLASH MRI. Magn. Reson. Med..

[bib58] Helms G., Dathe H., Weiskopf N., Dechent P. (2011). Identification of signal bias in the variable flip angle method by linear display of the algebraic ernst equation. Magn. Reson. Med..

[bib59] Helms G., Draganski B., Frackowiak R., Ashburner J., Weiskopf N. (2009). Improved segmentation of deep brain grey matter structures using magnetization transfer (MT) parameter maps. Neuroimage.

[bib60] Helms G., Hagberg G.E. (2009). In vivo quantification of the bound pool *T*_1_ in human white matter using the binary spin–bath model of progressive magnetization transfer saturation. Phys. Med. Biol..

[bib61] Henkelman R.M., Stanisz G.J., Graham S.J. (2001). Magnetization transfer in MRI: a review. NMR Biomed..

[bib62] Heule R., Ganter C., Bieri O., May (2015). Variable flip angle *T*_1_ mapping in the human brain with reduced s *t*_2_ ensitivity using fast radiofrequency-spoiled gradient echo imaging. Magn. Reson. Med..

[bib63] Hoult D.I. (2000). The principle of reciprocity in signal strength calculations - a mathematical guide. Concepts Magn. Reson. Part A Bridg. Educ. Res..

[bib64] Kamman R.L., Go K.G., Brouwer W., Berendsen H.J. (1988). Nuclear magnetic resonance relaxation in experimental brain edema: effects of water concentration, protein concentration, and temperature. Magn. Reson. Med..

[bib65] Kaneoke Y., Furuse M., Inao S., Saso K., Yoshida K., Motegi Y., Mizuno M., Izawa A. (1987). Spin-lattice relaxation times of bound water–its determination and implications for tissue discrimination. Magn. Reson. Imaging.

[bib66] Klein A., Ghosh S.S., Avants B., Yeo B.T.T., Fischl B., Ardekani B., Gee J.C., Mann J.J., Parsey R.V. (2010). Evaluation of volume-based and surface-based brain image registration methods. Neuroimage.

[bib67] Koenig S.H., Brown R.D., Ugolini R. (1993). A unified view of relaxation in protein solutions and tissue, including hydration and magnetization transfer. Magn. Reson. Med..

[bib68] Lambert C., Lutti A., Helms G., Frackowiak R., Ashburner J. (2013). Multiparametric brainstem segmentation using a modified multivariate mixture of Gaussians. Neuroimage: Clinica.

[bib69] Lee J., Nam Y., Choi J.Y., Kim E.Y., Oh S.-H., Kim D.-H. (2016). Mechanisms of T2* anisotropy and gradient echo myelin water imaging. NMR Biomed..

[bib70] Lee J.E., Chung M.K., Lazar M., DuBray M.B., Kim J., Bigler E.D., Lainhart J.E., Alexander A.L. (2009). A study of diffusion tensor imaging by tissue-specific, smoothing-compensated voxel-based analysis. Neuroimage.

[bib71] Lee Y., Callaghan M.F., Acosta-Cabronero J., Lutti A., Nagy Z. (2018). Establishing intra- and inter-vendor reproducibility of T1 relaxation time measurements with 3T MRI. Magn. Reson. Med..

[bib72] Lee Y., Callaghan M.F., Nagy Z. (2017). Analysis of the precision of variable flip angle T1 mapping with emphasis on the noise propagated from RF transmit field maps. Front. Neurosci..

[bib73] Liberman G., Louzoun Y., Ben Bashat D. (2013). T1 mapping using variable flip angle SPGR data with flip angle correction. J. Magn. Reson. Imag..

[bib74] Lorio S., Fresard S., Adaszewski S., Kherif F., Chowdhury R., Frackowiak R., Ashburner J., Helms G., Weiskopf N., Lutti A., Draganski B. (2016). New tissue priors for improved automated classification of subcortical brain structures on MRI. Neuroimage.

[bib75] Lorio S., Kherif F., Ruef A., Melie-Garcia L., Frackowiak R., Ashburner J., Helms G., Lutti A., Draganski B. (2016). Neurobiological origin of spurious brain morphological changes: a quantitative MRI study. Hum. Brain Mapp..

[bib76] Lorio S., Lutti A., Kherif F., Ruef A., Dukart J., Chowdhury R., Frackowiak R., Ashburner J., Helms G., Weiskopf N., Draganski B. (2014). Disentangling in vivo the effects of iron content and atrophy on the ageing human brain. Neuroimage.

[bib77] Lorio S., Tierney T.M., McDowell A., Arthurs O.J., Lutti A., Weiskopf N., Carmichael D.W. (2018). Flexible proton density (PD) mapping using multi-contrast variable flip angle (VFA) data. Neuroimage.

[bib78] Lutti A., Dick F., Sereno M.I., Weiskopf N. (2014). Using high-resolution quantitative mapping of R1 as an index of cortical myelination. Neuroimage.

[bib79] Lutti A., Hutton C., Finsterbusch J., Helms G., Weiskopf N. (2010). Optimization and validation of methods for mapping of the radiofrequency transmit field at 3T. Magn. Reson. Med..

[bib80] Lutti A., Hutton C., Weiskopf N. (2009). Optimization of 3D EPI technique for radio frequency (B1) field mapping at 3T. Proceedings of ISMRM 2009.

[bib81] Lutti A., Stadler J., Josephs O., Windischberger C., Speck O., Bernarding J., Hutton C., Weiskopf N. (2012). Robust and fast whole brain mapping of the RF transmit field B1 at 7T. PLoS One.

[bib82] Lutti A., Weiskopf N. (2013). Optimizing the accuracy of T1 mapping accounting for RF non-linearities and spoiling characteristics in FLASH imaging. Proceedings of ISMRM 2013.

[bib83] Marques J.P., Khabipova D., Gruetter R. (2017). Studying cyto and myeloarchitecture of the human cortex at ultra-high field with quantitative imaging: R1, R2* and magnetic susceptibility. Neuroimage.

[bib84] Mazziotta J., Toga A., Evans A., Fox P., Lancaster J., Zilles K., Woods R., Paus T., Simpson G., Pike B. (2001). A four-dimensional probabilistic atlas of the human brain. J. Am. Med. Inf. Assoc..

[bib85] Mazziotta J., Toga A., Evans A., Fox P., Lancaster J., Zilles K., Woods R., Paus T., Simpson G., Pike B. (2001). A probabilistic atlas and reference system for the human brain: international consortium for brain mapping (ICBM). Philos. Trans. R. Soc. B Biol. Sci..

[bib86] Mazziotta J.C., Toga A.W., Evans A., Fox P., Lancaster J. (1995). A probabilistic atlas of the human brain: theory and rationale for its development: the international consortium for brain mapping (ICBM). Neuroimage.

[bib87] Mezer A., Rokem A., Berman S., Hastie T., Wandell B.A. (2016). Evaluating quantitative proton-density-mapping methods. Hum. Brain Mapp..

[bib88] Mohammadi S., Callaghan M.F., Cercignani (2018).

[bib89] Mohammadi S., Carey D., Dick F., Diedrichsen J., Sereno M.I., Reisert M., Callaghan M.F., Weiskopf N. (2015). Whole-brain in-vivo measurements of the axonal g-ratio in a group of 37 healthy volunteers. Front. Neurosci..

[bib90] Mohammadi S., Keller S.S., Glauche V., Kugel H., Jansen A., Hutton C., Flöel A., Deppe M. (2012). The influence of spatial registration on detection of cerebral asymmetries using voxel-based statistics of fractional anisotropy images and tbss. PLoS One.

[bib91] Mohammadi S., Tabelow K., Ruthotto L., Feiweier T., Polzehl J., Weiskopf N. (2014). High-resolution diffusion kurtosis imaging at 3T enabled by advanced post-processing. Front. Neurosci..

[bib92] Mugler J.P., Brookeman J.R. (1990). Three-dimensional magnetization-prepared rapid gradient-echo imaging (3D MP RAGE). Magn. Reson. Med..

[bib93] Oh S.-H., Kim Y.-B., Cho Z.-H., Lee J. (2013). Origin of B0 orientation dependent R2* (=1/T2*) in white matter. Neuroimage.

[bib94] Papp D., Callaghan M.F., Meyer H., Buckley C., Weiskopf N. (2016). Correction of inter-scan motion artifacts in quantitative R1 mapping by accounting for receive coil sensitivity effects. Magn. Reson. Med..

[bib95] Pohmann R., Scheffler K. (2013). A theoretical and experimental comparison of different techniques for B_1_mapping at very high fields. NMR Biomed..

[bib96] Polzehl J., Tabelow K. (2016). Low SNR in diffusion MRI models. J. Am. Stat. Assoc..

[bib97] Preibisch C., Deichmann R. (2009). Influence of RF spoiling on the stability and accuracy of T1 mapping based on spoiled FLASH with varying flip angles. Magn. Reson. Med..

[bib98] Pruessmann K.P., Weiger M., Scheidegger M.B., Boesiger P. (1999). SENSE: sensitivity encoding for fast MRI. Magn. Reson. Med..

[bib99] Reisert M., Mader I., Umarova R., Maier S., Tebartz van Elst L., Kiselev V.G. (2013). Fiber density estimation from single q-shell diffusion imaging by tensor divergence. Neuroimage.

[bib100] Reuter M., Tisdall M.D., Qureshi A., Buckner R.L., van der Kouwe A.J., Fischl B. (2015). Head motion during MRI acquisition reduces gray matter volume and thickness estimates. Neuroimage.

[bib101] Ridgway G.R., Henley S.M.D., Rohrer J.D., Scahill R.I., Warren J.D., Fox N.C. (2008). Ten simple rules for reporting voxel-based morphometry studies. Neuroimage.

[bib102] Rosen A.F., Roalf D.R., Ruparel K., Blake J., Seelaus K., Villa L.P., Ciric R., Cook P.A., Davatzikos C., Elliott M.A., de La Garza A.G., Gennatas E.D., Quarmley M., Schmitt J.E., Shinohara R.T., Tisdall M.D., Craddock R.C., Gur R.E., Gur R.C., Satterthwaite T.D. (2018). Quantitative assessment of structural image quality. Neuroimage.

[bib103] Ruthotto L., Mohammadi S., Heck C., Modersitzki J., Weiskopf N., Meinzer H.-P., Deserno T.M., Handels H., Tolxdorff T. (2013). Hyperelastic susceptibility artifact correction of DTI in SPM. Bildverarbeitung für die Medizin 2013.

[bib104] Schabel M.C., Morrell G.R. (2008). Uncertainty in T1 mapping using the variable flip angle method with two flip angles. Phys. Med. Biol..

[bib105] Seif M., Leutritz T., Samson R.S., Curt A., Wheeler-Kingshott C.A.G., Freund P., Weiskopf N. (2018). A multi-center study on fast full-brain quantitative multi-parameter mapping of R1, MT, and R2*: scan-rescan repeatability and inter-site reproducibility. Proceedings of ISMRM 2018.

[bib106] Sereno M.I., Lutti A., Weiskopf N., Dick F. (2013). Mapping the human cortical surface by combining quantitative T1 with retinotopy. Cerebr. Cortex.

[bib107] Shuter B., Wang S.C., Roche J., Briggs G., Pope J.M. (1998). Relaxivity of Gd-EOB-DTPA in the normal and biliary obstructed Guinea pig. J. Magn. Reson. Imag..

[bib108] Smith S.M., Jenkinson M., Johansen-Berg H., Rueckert D., Nichols T.E., Mackay C.E., Watkins K.E., Ciccarelli O., Cader M.Z., Matthews P.M., Behrens T.E.J. (2006). Tract-based spatial statistics: voxelwise analysis of multi-subject diffusion data. Neuroimage.

[bib109] Soares J.M., Marques P., Alves V., Sousa N. (2013). A hitchhiker's guide to diffusion tensor imaging. Front. Neurosci..

[bib110] Sotiropoulos S.N., Jbabdi S., Xu J., Andersson J.L., Moeller S., Auerbach E.J., Glasser M.F., Hernandez M., Sapiro G., Jenkinson M., Feinberg D.A., Yacoub E., Lenglet C., Van Essen D.C., Ugurbil K., Behrens T.E.J., WU-Minn HCP Consortium (2013). Advances in diffusion MRI acquisition and processing in the human connectome project. Neuroimage.

[bib111] Steiger T.K., Weiskopf N., Bunzeck N. (2016). Iron level and myelin content in the ventral striatum predict memory performance in the aging brain. J. Neurosci..

[bib112] Stikov N., Campbell J.S.W., Stroh T., Lavelée M., Frey S., Novek J., Nuara S., Ho M.-K., Bedell B.J., Dougherty R.F., Leppert I.R., Boudreau M., Narayanan S., Duval T., Cohen-Adad J., Picard P.-A., Gasecka A., Côté D., Pike G.B. (2015). In vivo histology of the myelin g-ratio with magnetic resonance imaging. Neuroimage.

[bib113] Stikov N., Perry L.M., Mezer A., Rykhlevskaia E., Wandell B.A., Pauly J.M., Dougherty R.F. (2011). Bound pool fractions complement diffusion measures to describe white matter micro and macrostructure. Neuroimage.

[bib114] Stüber C., Morawski M., Schäfer A., Labadie C., Wähnert M., Leuze C., Streicher M., Barapatre N., Reimann K., Geyer S., Spemann D., Turner R. (2014). Myelin and iron concentration in the human brain: a quantitative study of MRI contrast. Neuroimage.

[bib115] Tabelow K., D'Alonzo C., Polzehl J., F.Callaghan M., Ruthotto L., Weiskopf N., Mohammadi S. (2016). How to achieve very high resolution quantitative MRI at 3T. Proceedings of HBM 2016.

[bib116] Tabelow K., D'Alonzo C., Ruthotto L., Callaghan M.F., Weiskopf N., Polzehl J., Mohammadi S. (2017). Removing the estimation bias due to the noise floor in multi-parameter maps. Proceedings of ISMRM 2017.

[bib117] Tabelow K., Mohammadi S., Weiskopf N., Polzehl J. (2015). POAS4SPM: a toolbox for SPM to denoise diffusion MRI data. Neuroinformatics.

[bib118] Thompson P., Toga A.W. (1996). A surface-based technique for warping three-dimensional images of the brain. IEEE Trans. Med. Imag..

[bib119] Trampel R., Bazin P.-L., Pine K., Weiskopf N. (2017). In-vivo magnetic resonance imaging (MRI) of laminae in the human cortex. Neuroimage.

[bib120] Volz S., Nöth U., Jurcoane A., Ziemann U., Hattingen E., Deichmann R. (2012). Quantitative proton density mapping: correcting the receiver sensitivity bias via pseudo proton densities. Neuroimage.

[bib121] Volz S., Nöth U., Rotarska-Jagiela A., Deichmann R. (2010). A fast B1-mapping method for the correction and normalization of magnetization transfer ratio maps at 3T. Neuroimage.

[bib122] Wang H.Z., Riederer S.J., Lee J.N. (1987). Optimizing the precision in T1 relaxation estimation using limited flip angles. Magn. Reson. Med..

[bib123] Weiskopf N., Callaghan M.F., Josephs O., Lutti A., Mohammadi S. (2014). Estimating the apparent transverse relaxation time (R2(*)) from images with different contrasts (ESTATICS) reduces motion artifacts. Front. Neurosci..

[bib124] Weiskopf N., Helms G. (2008). Multi-parameter mapping of the human brain at 1mm resolution in less than 20 minutes. Proceedings of ISMRM 2008.

[bib125] Weiskopf N., Lutti A., Helms G., Novak M., Ashburner J., Hutton C. (2011). Unified segmentation based correction of R1 brain maps for RF transmit field inhomogeneities (UNICORT). Neuroimage.

[bib126] Weiskopf N., Mohammadi S., Lutti A., Callaghan M.F. (2015). Advances in MRI-based computational neuroanatomy. Curr. Opin. Neurol..

[bib127] Weiskopf N., Suckling J., Williams G., Correia M.M., Inkster B., Tait R., Ooi C., Bullmore E.T., Lutti A. (2013). Quantitative multi-parameter mapping of R1, PD(*), MT, and R2(*) at 3T: a multi-center validation. Front. Neurosci..

[bib128] Wharton S., Bowtell R. (2012). Fiber orientation-dependent white matter contrast in gradient echo MRI. Proc. Natl. Acad. Sci. U.S.A..

[bib129] Whitaker K.J., Vértes P.E., Romero-Garcia R., Váša F., Moutoussis M., Prabhu G., Weiskopf N., Callaghan M.F., Wagstyl K., Rittman T. (2016). Adolescence is associated with genomically patterned consolidation of the hubs of the human brain connectome. Proc. Natl. Acad. Sci. U.S.A..

[bib130] Wood T. (2018). QUIT: QUantitative imaging tools. J. Open Source Softw..

[bib131] Yablonskiy D.A. (1998). Quantitation of intrinsic magnetic susceptibility-related effects in a tissue matrix. phantom study. Magn. Reson. Med..

[bib132] Yarnykh V.L. (2007). Actual flip-angle imaging in the pulsed steady state: a method for rapid three-dimensional mapping of the transmitted radiofrequency field. Magn. Reson. Med..

[bib133] Yarnykh V.L. (2010). Optimal radiofrequency and gradient spoiling for improved accuracy of T1 and B1 measurements using fast steady-state techniques. Magn. Reson. Med..

[bib134] Yendiki A., Panneck P., Srinivasan P., Stevens A., Zöllei L., Augustinack J., Wang R., Salat D., Ehrlich S., Behrens T., Jbabdi S., Gollub R., Fischl B. (2011). Automated probabilistic reconstruction of white-matter pathways in health and disease using an atlas of the underlying anatomy. Front. Neuroinf..

[bib135] Ziegler G., Grabher P., Thompson A., Altmann D., Hupp M., Ashburner J., Friston K., Weiskopf N., Curt A., Freund P. (2018). Progressive neurodegeneration following spinal cord injury. Neurology.

